# ZNF143 is a transcriptional regulator of nuclear-encoded mitochondrial genes that acts independently of looping and CTCF

**DOI:** 10.1016/j.molcel.2024.11.031

**Published:** 2025-01-02

**Authors:** Mikhail D. Magnitov, Michela Maresca, Noemí Alonso Saiz, Hans Teunissen, Jinhong Dong, Kizhakke M. Sathyan, Luca Braccioli, Michael J. Guertin, Elzo de Wit

**Affiliations:** 1 Division of Gene Regulation, The Netherlands Cancer Institute, Plesmanlaan 121, 1066 CX Amsterdam, the Netherlands; 2 Department of Clinical Genetics, Erasmus University MC, Dr. Molewaterplein 40, 3015 GD Rotterdam, the Netherlands; 3 Center for Cell Analysis and Modeling, University of Connecticut, 400 Farmington Avenue, Farmington, CT, USA; 4 Department of Genetics and Genome Sciences, University of Connecticut, 400 Farmington Avenue, Farmington, CT, USA

**Keywords:** gene regulation, transcription, mitochondria, development, 3D genome, chromatin looping, differentiation

## Abstract

Gene expression is orchestrated by transcription factors, which function within the context of a three-dimensional genome. Zinc-finger protein 143 (ZNF143/ZFP143) is a transcription factor that has been implicated in both gene activation and chromatin looping. To study the direct consequences of ZNF143/ZFP143 loss, we generated a ZNF143/ZFP143 depletion system in mouse embryonic stem cells. Our results show that ZNF143/ZFP143 degradation has no effect on chromatin looping. Systematic analysis of ZNF143/ZFP143 occupancy data revealed that a commonly used antibody cross-reacts with CTCF, leading to its incorrect association with chromatin loops. Nevertheless, ZNF143/ZFP143 specifically activates nuclear-encoded mitochondrial genes, and its loss leads to severe mitochondrial dysfunction. Using an *in vitro* embryo model, we find that ZNF143/ZFP143 is an essential regulator of organismal development. Our results establish ZNF143/ZFP143 as a conserved transcriptional regulator of cell proliferation and differentiation by safeguarding mitochondrial activity.

## Highlights

• Acute degradation of ZFP143 leads to rapid and specific loss of gene transcription
• Consequences of ZFP143 loss are inconsistent with it being a general looping factor
• ZFP143 is a conserved transcriptional regulator of nuclear-encoded mitochondrial genes
• ZFP143 regulation of mitochondrial homeostasis is crucial for multicellular development

## Graphical abstract

**Figure:**
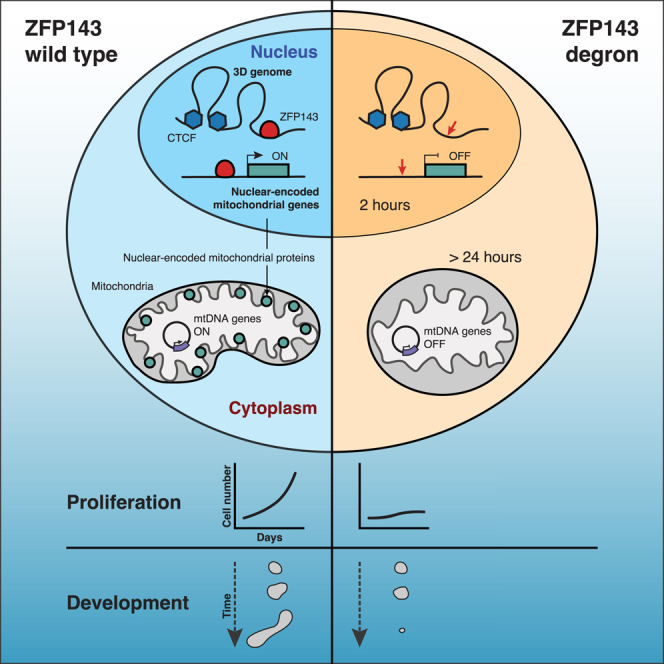

## Teaser

ZNF143 has been implicated in gene activation and chromatin looping. Magnitov et al. show that ZNF143 has no general looping factor activity and explain why this function has been misattributed. Instead, ZNF143 regulates transcription of nuclear-encoded mitochondrial genes, ensuring energy homeostasis, proliferation, and differentiation potential of stem cells.

## Introduction

Gene expression is a key biological process that is regulated by transcription factors (TFs), which recognize and bind specific DNA sequences.^1^ Some TFs act by binding directly to gene promoters, while others modulate expression by targeting distal enhancers, which can be located tens or even hundreds of kilobases from their target promoters.^2^^,^^3^^,^^4^

Zinc-finger protein 143 (ZNF143/ZFP143, referring to the human/mouse proteins, respectively) is a sequence-specific DNA-binding TF. It is an ortholog of selenocysteine tRNA gene transcription activating factor (Staf) originally identified in *Xenopus laevis*.^5^^,^^6^^,^^7^ Staf was first described as a transcriptional activator of small nuclear RNA (snRNA) genes transcribed by RNA polymerases II and III^8^ and was later shown to also be capable of activating transcription from an mRNA promoter.^7^^,^^9^ Transcriptional activation by Staf is mediated by recognition of its consensus DNA-binding motif, the Staf binding site (SBS).^5^^,^^8^

Genome-wide analyses in mammalian cells revealed that SBS motifs are present in thousands of promoters and bound by ZNF143,^10^^,^^11^^,^^12^ which directly regulates the expression of its target genes.^12^^,^^13^ ZNF143 is expressed in a wide range of tissues^14^ and was identified as an “essential” gene in genome-wide genetic screens.^15^^,^^16^ Given its established transcriptional regulator role, ZNF143 has been implicated in many biological, pathological, and developmental processes, such as proliferation and cell-cycle regulation,^12^^,^^17^^,^^18^^,^^19^^,^^20^ cancer cell survival,^18^^,^^20^^,^^21^^,^^22^ embryonic development,^23^^,^^24^^,^^25^^,^^26^ and many others.^27^^,^^28^

Recent studies have suggested that ZNF143 is also involved in genome organization. Two key players in the organization of chromosomes are the cohesin complex, which binds to and extrudes DNA to create chromatin loops, and CCCTC-binding factor (CTCF), which creates extrusion barriers by blocking cohesin.^29^ Multiple studies reported that ZNF143 co-localizes with CTCF^30^^,^^31^^,^^32^^,^^33^^,^^34^^,^^35^^,^^36^^,^^37^^,^^38^^,^^39^ and participates in chromatin looping.^33^^,^^34^^,^^35^^,^^36^^,^^37^^,^^38^^,^^39^^,^^40^^,^^41^^,^^42^^,^^43^^,^^44^^,^^45^^,^^46^^,^^47^ Deletion or silencing of ZNF143 resulted in a weakening of a subset of chromatin interactions, particularly those centered on promoter and enhancer regions.^34^^,^^38^^,^^39^^,^^46^^,^^47^ Based on these experiments, ZNF143 has been considered an important general looping factor besides CTCF and cohesin. It has been specifically suggested that ZNF143 may function in conjunction with CTCF to establish enhancer-promoter loops.^34^^,^^38^^,^^39^^,^^47^ However, these studies were performed in a non-acute depletion setting, making it difficult to disentangle the direct and indirect effects of ZNF143 loss on chromatin structure.

In this work, we aimed to understand the direct role of ZNF143 in chromatin looping and the interplay with transcriptional regulation. We used an acute protein degradation system to deplete ZFP143 from mouse embryonic stem cells (mESCs). By measuring chromatin occupancy and chromatin interactions, we were unable to establish a general role for ZFP143 in chromatin looping. Systematic re-analysis of publicly available ZNF143 binding datasets revealed antibody cross-reactivity with CTCF, confirmed by our chromatin immunoprecipitation sequencing (ChIP-seq) experiments in a ZNF143 depletion background. We propose that the described antibody cross-reactivity with CTCF led to the erroneous association of ZNF143 with chromatin looping. These results rule out ZNF143 as a general looping factor. Using nascent transcriptome profiling, we find that ZFP143 acts as a transcriptional regulator of nuclear-encoded mitochondrial genes, particularly of the mitochondrial ribosome and oxidative phosphorylation complexes. Loss of ZFP143 leads to their immediate downregulation at transcriptome and proteome levels, inducing a mitochondrial dysfunction phenotype. Our analyses establish ZNF143 as a conserved transcriptional regulator of cellular proliferation and differentiation, which acts independently of chromatin looping and CTCF.

## Results

### Establishment of ZFP143 degron cell line

**Figure 1:**
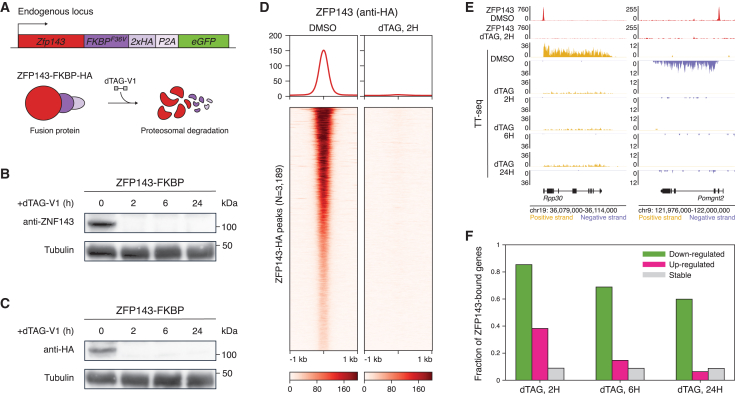
Generation and validation of a system for rapid depletion of ZFP143 in mESCs (A) Schematic representation of the dTAG degron system. The endogenous *Zfp143* locus is modified to create a fusion with the FKBP12^F36V^-2xHA-P2A-eGFP cassette. (B) Western blot analysis of ZFP143 levels with an antibody against the endogenous ZFP143 at different time points after dTAG-V1 treatment. (C) Western blot analysis of ZFP143 levels with an antibody against the HA tag at different time points after dTAG-V1 treatment. (D) Tornado plots showing ZFP143-HA ChIP-seq signal centered at ZFP143-HA peaks in DMSO- and dTAG-V1-treated cells. (E) Genomic tracks showing ZFP143-HA ChIP-seq (red) and TT-seq nascent transcription (yellow for sense and purple for antisense transcription) at *Rpp30* and *Pomgnt2* in DMSO-treated and dTAG-V1-treated cells. (F) Fraction of ZFP143-bound genes among downregulated (green), upregulated (pink), and stable (gray) genes in the TT-seq at different time points after ZFP143 depletion.

To investigate the direct impact of ZFP143 loss on chromatin structure and gene expression, we utilized the degradation tag (dTAG) system to rapidly deplete ZFP143 from mESCs.^48^ Using CRISPR-Cas9-mediated genome editing, we endogenously tagged the C-terminal domain of ZFP143 with the FKBP12^F36V^ domain, 2xHA tag and eGFP (Figure 1A; Table S1). We characterized the kinetics of ZFP143 loss by treating mESCs with the dTAG-V1 small molecule, which targets the fusion protein to the von Hippel-Lindau (VHL) E3 ubiquitin ligase.^49^ Immunoblotting showed that ZFP143 was depleted from mESCs within 2 h of dTAG-V1 treatment (Figures 1B and 1C). To further validate the depletion of ZFP143, we performed ChIP-seq using an antibody against the introduced HA tag. We found that ZFP143 was no longer detectable on chromatin after 2 h of dTAG-V1 treatment (Figure 1D), suggesting efficient depletion of the target protein.

ZFP143-HA peak annotation revealed that most of the detected peaks were located at active promoters^50^ (Figure S1A). *De novo* motif analysis revealed two top-scoring motifs, which corresponded to the known SBS1 and SBS2 sites^12^ and were present in 95% of ZFP143-HA peaks (Figures S1B–S1D), indicating that our ChIP-seq data specifically identifies ZFP143 binding. Comparing our ZFP143-HA ChIP-seq data with a previously published dataset generated using a custom antibody against ZFP143 in mESCs^12^ demonstrated a high degree of peak overlap and similarity in the binding profiles (Figures S1E–S1G), indicating that introduction of the FKBP12^F36V^ domain and HA tags do not noticeably impact chromatin occupancy of ZFP143 at its target sites.

To probe the effect of ZFP143 loss on transcription, we performed nascent transcript profiling using transient transcriptome sequencing (TT-seq) in control cells and after 2, 6, and 24 h of ZFP143 depletion.^51^ The majority of genes affected by ZFP143 loss are downregulated (false discovery rate [FDR] < 0.05, absolute log_2_ fold change > 0.5, Figures 1E and S1H), consistent with its role as a transcriptional activator. By combining chromatin occupancy with nascent transcription, we found that 85% of the genes that were downregulated after 2 h have ZFP143 binding at the promoter (STAR Methods; Figure 1F), indicating that these genes are direct targets of ZFP143. Our findings confirm the functional depletion of ZFP143 and highlight the reliability of our experimental system in detecting the direct effects of ZFP143 loss.

### Acute loss of ZFP143 is not consistent with a role in 3D genome organization

**Figure 2:**
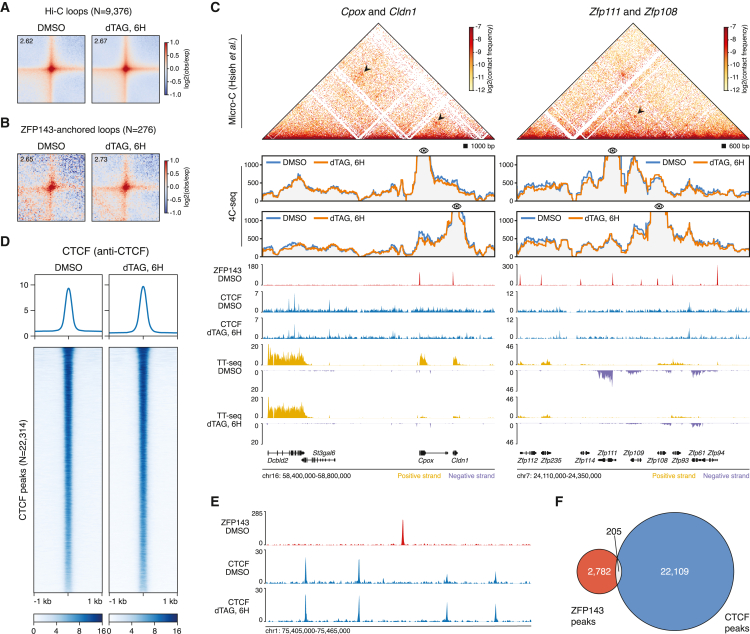
ZFP143 depletion has no detectable effect on 3D genome structure and CTCF binding (A) Average Hi-C loops^52^ in DMSO-treated and dTAG-V1-treated cells. Value in the upper-right corner indicates the interaction strength of the loop over the background. (B) Same as in (A), but for the average ZFP143-associated Hi-C loops. (C) 4C-seq data generated for the *Cpox* and *Cldn1* (left) and *Zfp111* and *Zfp108* (right) loci. The matrix in the top panel represents interaction frequencies in a previously published high-resolution Micro-C dataset.^54^ The arrows point to detected Micro-C chromatin loops. The bottom panel shows 4C contact profiles in DMSO-treated (blue) and dTAG-V1-treated (orange) cells. Genomic tracks show ZFP143-HA ChIP-seq (red), calibrated CTCF ChIP-seq (blue), TT-seq nascent transcription (yellow for sense and purple for antisense transcription) in DMSO-treated and dTAG-V1-treated cells. (D) Tornado plots of calibrated CTCF ChIP-seq signal centered at CTCF peaks in DMSO-treated and dTAG-V1-treated cells. (E) Genomic tracks showing ZFP143-HA ChIP-seq (red) in DMSO-treated cells and calibrated CTCF ChIP-seq (blue) in DMSO-treated and dTAG-V1-treated cells. (F) Venn diagram showing the overlap between ZFP143-HA (red) and CTCF (blue) peaks.

Recent studies have suggested a role for ZFP143 in 3D genome organization by contributing to chromatin loop formation.^34^^,^^38^^,^^39^^,^^46^^,^^47^ To determine the direct consequences of ZFP143 loss on chromatin looping, we performed *in situ* Hi-C following 6 h of ZFP143 depletion. Relative contact probability curves were almost indistinguishable, suggesting no overall changes in genome topology induced by ZFP143 depletion (Figure S2A). To determine whether chromatin loops are affected upon ZFP143 depletion, we performed a pile-up analysis of the loops previously identified in a high-resolution Hi-C mESC dataset.^52^ Surprisingly, we did not detect a decrease in genome-wide loop strength (Figure 2A). To investigate whether specific chromatin loop types were affected, we analyzed cohesin-mediated, enhancer-promoter, and promoter-promoter loops.^53^ Again, we did not detect changes in contact frequency for these loop classes (Figure S2B). Finally, to test whether chromatin loops associated with ZFP143 are affected, we analyzed loops that had ZFP143 binding at one or both anchors. Also, here, we were not able to detect any obvious changes in the interaction strength (Figures 2B and S2C).

Puzzled by these findings, we sought to improve the resolution by obtaining detailed interaction profiles for a few loci. We selected gene promoters that are bound by ZFP143, are downregulated within 2 h of ZFP143 depletion, and form a loop in a high-resolution Micro-C mESC dataset.^54^ We performed 4C-seq after 6 h of ZFP143 depletion, using the selected promoters as viewpoints.^55^ Similar to the genome-wide Hi-C results, the local 4C-seq interaction profiles were mostly unaffected by ZFP143 depletion for the tested promoters (Figures 2C and S2D). Overall, our comprehensive analyses do not detect systematic changes in the chromatin interaction landscape of mESCs following ZFP143 depletion. Therefore, our results are inconsistent with a role for ZFP143 as a general looping factor that establishes or maintains genome architecture.

### CTCF binding and distribution is unaffected upon ZFP143 depletion

Recently, a knockout of ZFP143 in mouse hematopoietic stem and progenitor cells suggested a loss of CTCF binding in the regions where ZFP143 and CTCF co-localize,^38^ implicating ZFP143 in positioning CTCF on chromatin. To determine whether we could detect a similar dependency, we performed calibrated CTCF ChIP-seq after 6 h of ZFP143 depletion. However, we could not detect any consistent change in CTCF occupancy (Figure 2D). Heatmaps of ZFP143 binding intensity at CTCF peaks demonstrated hardly any ZFP143 signal at these regions (Figure S2E). Moreover, very little CTCF signal was detected at the ZFP143 binding sites (Figure S2F). Visual inspection of the chromatin occupancy tracks indicated that CTCF and ZFP143 binding were almost mutually exclusive, and the calculated overlap between CTCF and ZFP143 peaks showed that only a minority of them were shared (Figures 2E and 2F). In contrast to previous reports, these results suggest that depletion of ZFP143 does not affect CTCF positioning and that ZFP143 and CTCF bind to distinct genomic regions.

### Antibody cross-reactivity mistakenly identified ZNF143 co-localization with CTCF

**Figure 3:**
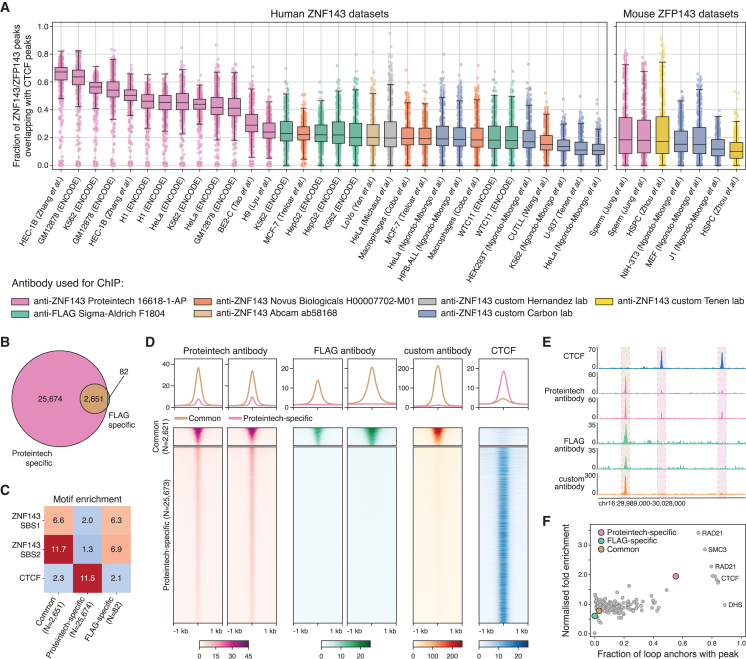
Re-analysis of publicly available ChIP-seq data reveals ZNF143 antibody cross-reactivity with CTCF (A) Overlap between ZNF143 peaks from re-analyzed publicly available data and CTCF peaks from CISTROME^56^ for human (left) and mouse (right) datasets. Each dot represents the overlap between the indicated ZNF143 peak set with an individual CTCF peak set. Colors represent the antibody used for chromatin immunoprecipitation. (B) Venn diagram showing the overlap between ZNF143 peaks detected by Proteintech (light pink) and FLAG (light green) antibodies in K562 cells. (C) Heatmap showing the enrichment of ZNF143 SBS and CTCF motifs in common, Proteintech-specific, and FLAG-specific peaks in K562 cells. (D) Tornado plots of ChIP-seq signals detected by Proteintech (light pink), FLAG (light green), and custom^12^ (orange) antibodies, and CTCF signal (blue) in K562 cells. The ChIP-seq signals are centered on common (top) and Proteintech-specific (bottom) peaks. (E) Genomic tracks showing ChIP-seq signals for CTCF (blue) and signals detected by Proteintech (pink), FLAG (light green), and custom^12^ (orange) antibodies in K562 cells. Rectangles indicate common (left) and Proteintech-specific (middle and right) peaks in the region. (F) Scatterplot of the percentage of loop anchors overlapping the peak (x axis) against the fold enrichment of peaks in loop anchors (y axis) for a number of DNA-binding proteins^40^ and for Proteintech-specific, FLAG-specific, and common peaks in K562 cells.

The results we obtained from our acute depletion of ZFP143 appear at odds with previous literature reports. To determine the source and nature of the discrepancies, we systematically re-analyzed published ZNF143 and CTCF ChIP-seq data. For each ZNF143 dataset, we calculated the proportion of peaks that overlapped with CTCF peak sets available in the CISTROME database^56^ (STAR Methods; Figure 3A). For the majority of ZNF143 datasets, the median proportion of peaks overlapping with CTCF peaks was approximately 20%. However, we found that for certain datasets the overlap percentage is much higher (>40%). The datasets that showed the greatest overlap with CTCF peaks were obtained using the same antibody, anti-ZNF143 Proteintech 16618-1-AP (hereafter referred to as Proteintech), a polyclonal antibody that recognizes endogenous ZNF143 protein. Most of these datasets originated from the ENCODE project, in which the overlap with CTCF was first reported.^30^

To further explore the overlap between ZNF143 and CTCF, we compared ChIP-seq data generated using the Proteintech antibody (anti-ZNF143) with C-terminally 3xFLAG-tagged ZNF143 ChIP-seq (anti-FLAG-ZNF143) in K562 cells.^57^ We found an almost 10-fold higher number of peaks detected by the Proteintech antibody, compared with the FLAG antibody (Figure 3B). Importantly, the anti-FLAG-ZNF143 peaks were an almost complete subset of the anti-ZNF143 peaks. Motif enrichment analysis on these peak categories showed that two ZNF143 SBS motifs were strongly enriched in the common and FLAG-specific categories but had low enrichment in the Proteintech-specific peaks, while the CTCF motif was highly enriched in the Proteintech-specific peaks and only slightly enriched in the other two categories of peaks (Figure 3C). Visual inspection and genome-wide quantification of the chromatin occupancy showed that the Proteintech anti-ZNF143 antibody detected signal at CTCF binding sites, whereas nothing was detected in the anti-FLAG-ZNF143 data at the same regions (Figures 3D and 3E). Additionally, data generated with a custom anti-ZNF143 antibody^12^ showed a similar binding profile to anti-FLAG-ZNF143, with strong binding at the common peaks and lack of binding at the CTCF-bound Proteintech-specific peaks (Figures 3D and 3E). Of note, the signal detected with the Proteintech antibody at the Proteintech-specific peaks has a much lower intensity, compared with its signal at the common peaks. These analyses strongly suggest that the data obtained with the anti-ZNF143 Proteintech antibody have an internal bias, due to antibody-cross reactivity with CTCF.

To directly investigate the potential cross-reactivity of the anti-ZNF143 Proteintech antibody with CTCF, we used a ZNF143-FKBP HEK293T cell line, in which ZNF143 has been tagged with FKBP12^F36V^ domain and 2xHA tag and can be acutely depleted by treatment with dTAG-V1.^58^ We generated ChIP-seq data using the Proteintech antibody in the presence and absence of ZNF143 and compared it with previously published ChIP-seq data generated using the HA antibody^58^ (Figure S3A). As expected, the Proteintech antibody detected more peaks, compared with the HA antibody, and these peaks showed a strong enrichment for CTCF motifs (Figures S3B and S3C). Chromatin occupancy tracks showed that Proteintech antibody yielded signal at CTCF binding sites, whereas no signal could be detected at the same regions for the HA antibody (Figure S3D). Most importantly, upon ZNF143 depletion, the signal detected by the Proteintech antibody at CTCF sites remained and became even stronger (Figure S3E), suggesting that the binding signal is independent of ZNF143. These results confirm the cross-reactivity of the Proteintech antibody with CTCF.

Systematic analysis of 3D genome organization using *in situ* Hi-C previously revealed that ZNF143, together with CTCF and cohesin subunits, is highly enriched at chromatin loop anchors in GM12878 and K562 cells.^40^ We recalculated the enrichments of proteins at the loop anchors defined in the K562 Hi-C data, using the three ZNF143 peak categories mentioned above. Only the Proteintech-specific peaks showed a high enrichment at loop anchors, while the common and FLAG-specific categories do not (Figure 3F). Therefore, the reported enrichment of ZNF143 at chromatin loop anchors can be explained by the signal at the CTCF binding sites caused by the cross-reactivity described above. These analyses further challenge the idea that ZNF143 has a role as a general looping factor.

Finally, several studies reported an interdependence between ZNF143 and CTCF, namely, two opposing claims that CTCF is positioned on chromatin by ZNF143^38^ and that ZNF143 occupancy depends on CTCF at regions where the two proteins bind together.^39^ Re-analysis of the data from these studies suggests that the reduction of CTCF on chromatin after ZNF143 knockout may have been caused by GC bias in the ChIP-seq data (STAR Methods; Figures S4A–S4F), whereas the reduction of ZNF143 occupancy following CTCF depletion can be explained by the use of the Proteintech antibody (STAR Methods; Figures S4G and S4H). In addition, we found that all ZNF143-CTCF motif pairs, reported to localize 37 bp apart from each other,^38^ are contained within SINE/B2 repetitive elements and are likely to be an artifact (STAR Methods; Figures S4I and S4J). Taken together, these results suggest that cooperation between ZNF143 and CTCF is highly unlikely.

### ZNF143 target genes are conserved across cell types and organisms

Given that ZFP143 is unlikely to be involved in chromatin looping, we focused on its role as a TF. Since ZFP143 binds predominantly to transcription start sites, we used the ZFP143-HA ChIP-seq data to identify ZFP143-bound gene promoters in mESCs (*N =* 2,226). Gene Ontology analysis of ZFP143-bound genes revealed that they belong to functional categories previously associated with ZFP143,^12^ such as snRNAs, cell cycle, and metabolism (Figure S5A). Additionally, we found gene sets involved in translation, mitochondrial functions, and stress-related pathways. To understand the conservation of ZFP143-bound genes, we utilized the systematically re-analyzed ZNF143 ChIP-seq data. We identified a set of ZNF143 binding sites conserved across human and mouse cells and associated them to gene promoters (1,696 and 1,504 conserved ZNF143-bound genes in human and mouse, respectively; STAR Methods; Figure S5B). Overlapping ZFP143-bound genes from our study with conserved ZNF143-bound genes in human and mouse revealed a strong overlap between the genes (54% and 63% for conserved human and mouse ZNF143-bound genes, respectively) and the functional categories to which they belong (Figures S5C–S5E). This suggests that regulation by ZNF143 is highly conserved across cell types and organisms.

### ZFP143 regulates nuclear genes encoding mitochondrial proteins

**Figure 4:**
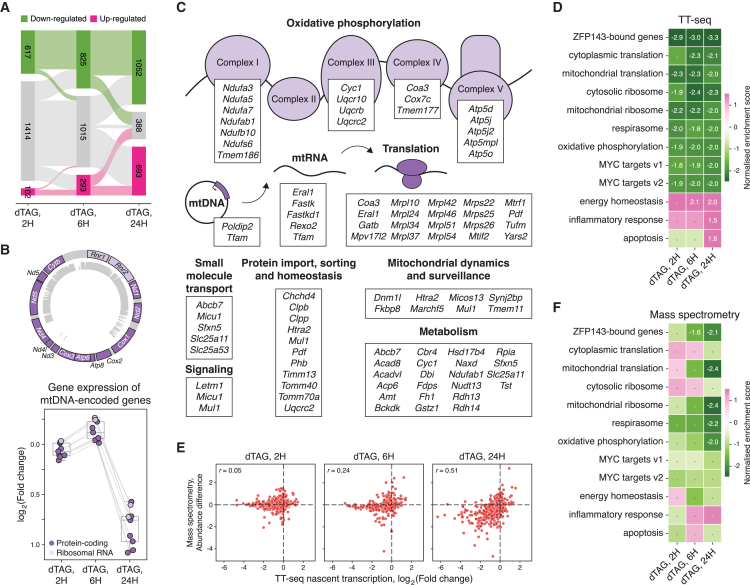
ZFP143 depletion leads to downregulation of mitochondria-related genes at transcriptome and proteome levels (A) Sankey diagram showing the differentially expressed genes following ZFP143 depletion (upregulated genes in pink, downregulated genes in green). (B) Schematic of the genes and single-read mappability^59^ (k = 36) for mitochondrial DNA (top) and fold changes of the mitochondrial-encoded genes detected by TT-seq following ZFP143 depletion (bottom). (C) Schematic of ZFP143-bound nuclear-encoded mitochondrial genes that are downregulated in TT-seq data. Genes are grouped by mitochondrial function or protein complex as defined in the MitoCarta database.^60^ (D) Gene set enrichment analysis of TT-seq data after ZFP143 depletion (upregulated gene sets in pink, downregulated gene sets in green). The normalized enrichment score for significant gene sets (FDR < 0.05) is shown. (E) Correlation of nascent RNA production (x axis) and protein abundance (y axis) for ZFP143-bound genes following ZFP143 depletion. Values in the upper-left corners indicate Pearson correlation coefficients. (F) Gene set enrichment analysis of mass spectrometry data after ZFP143 depletion (upregulated gene sets in pink, downregulated gene sets in green). The normalized enrichment score for significant gene sets (FDR < 0.05) is shown.

To analyze the direct functional consequences of ZFP143 loss, we performed a more detailed analysis of our TT-seq dataset following ZFP143 depletion. Differential expression analysis identified 617 downregulated genes, the number of which increased to 1,052 with longer depletion times (FDR < 0.05, absolute log_2_ fold change > 0.5, Figure 4A). In the first 6 h following ZFP143 depletion, 293 genes increased their expression, whereas after 24 h, we observed 693 upregulated genes (Figure 4A). The lack of ZFP143 binding to the promoters of the upregulated genes (Figure 1F) and the late response indicate that the effect of ZFP143 depletion on the expression of these genes is indirect. Of note, not all ZFP143-bound genes are downregulated, suggesting that ZFP143 directly and exclusively controls only a subset of these.

Inspection of differentially transcribed genes revealed that all protein-coding and rRNA genes from the mitochondrial genome, detected by TT-seq, showed significant downregulation after 24 h of ZFP143 loss (Figure 4B). Consistent with this finding, the essential mitochondrial TF *Tfam* is downregulated after 2 h of dTAG-V1 treatment and is a target of ZFP143.^61^ To investigate whether other nuclear-encoded mitochondrial-related genes are downregulated upon ZFP143 loss, we annotated differentially expressed genes using the MitoCarta database.^60^ We found multiple examples that show similar behavior, including subunits of oxidative phosphorylation complexes *Ndufab1*, *Cyc1*, and *Coa3*; translation factors *Mtrf1* and *Tufm*; mitochondrial fission regulator *Dnm1l*; and others^62^^,^^63^ (Figure 4C). To gain insight into which gene categories are deregulated upon ZFP143 depletion, we performed gene set enrichment analysis (GSEA) on the TT-seq data, which revealed that genes involved in cytoplasmic and mitochondrial translation, oxidative phosphorylation components, and MYC targets show significant downregulation (Figure 4D). These gene sets were also identified as ZFP143-bound genes in our analyses (Figures S5A and S5E). Hardly any gene sets showed significant upregulation at the early time points, although after 24 h of ZFP143 depletion, differentiation-related gene sets as well as apoptosis and inflammatory response categories show significantly increased expression (Figure 4D), suggesting activation of a general stress response in mESCs.

To investigate how transcriptional changes translate into changes at the protein level, we performed quantitative mass spectrometry-based proteome analysis. As a control, we first treated untagged parental mESCs with dTAG-V1 and identified only minimal changes in the proteome, indicating that dTAG-V1 treatment does not appreciably affect protein abundance (Figure S6A). In ZFP143-depleted mESCs, the composition of the proteome was largely unaffected at 2 and 6 h after depletion. However, at 24 h after ZFP143 depletion, the levels of hundreds of proteins were significantly reduced, 73% of which are encoded by ZFP143-bound genes (Student’s t test −log_10_(*p* value) ≥ 1.3, absolute abundance difference ≥ 0.5, Figures S6B and S6C), suggesting a direct relationship between gene regulation by ZFP143 and the abundance of gene products in the proteome. To assess the temporal relationship between transcription and protein levels, we compared the changes in nascent RNA production and protein abundance. For ZFP143-bound genes, the decrease in nascent transcription shows strong correlation with a decrease in protein abundance only after 24 h of ZFP143 loss (Pearson r = 0.51, *p* < 2.2 × 10^−16^, Figure 4E). Additionally, GSEA revealed that after 24 h of ZFP143 depletion, mitochondrial translation and oxidative phosphorylation functional categories become significantly downregulated in the proteome (Figure 4F). This indicates that ZFP143-bound genes that are downregulated in transcription at early time points are manifested in the proteome at later time points, consistent with delayed functional consequences.

To address the long-term effects of ZFP143 depletion on the transcriptome, we also performed RNA sequencing (RNA-seq) after 24, 48, and 72 h of ZNF143 depletion. We identified 440 differentially expressed genes after 24 h, of which 338 were downregulated (Figures S7A and S7B). This number increased to 1,514 and 2,290 genes after 48 and 72 h of ZFP143 depletion, respectively, and was roughly equally divided between upregulated and downregulated genes. Similar to the TT-seq, in the mRNA pool, downregulated, but not upregulated, genes were predominantly ZFP143 targets (Figure S7C). Direct comparison of the differentially expressed genes in the nascent RNA and in the stable mRNA pool showed a high concordance between the two, especially at the 24-h time point (Figures S7D and S7E). Consistent with the nascent transcriptome, after 24 h of ZFP143 depletion translation, oxidative phosphorylation and MYC targets gene sets were also downregulated in the mRNA pool (Figure S7F). In addition, other energy-related gene sets, such as glycolysis and fatty acid metabolism, and cell-cycle-related gene sets, such as E2F targets and G2M checkpoint, were found to be downregulated at 48 and 72 h after depletion (Figure S7F). In contrast, several signaling pathways were upregulated at the later time points (Figure S7F). The delayed transcriptional response of these gene sets, and the fact that these changes are not seen in the nascent transcriptome, suggests that these mRNA alterations are not a primary transcriptional outcome but are secondary effects induced to adapt to the effects of ZFP143 loss. Taken together, these results show that ZFP143 is a transcriptional regulator of nuclear-encoded mitochondrial genes, and its depletion has an immediate effect on their transcription, mRNA, and protein levels.

### Prolonged ZFP143 loss leads to decreased cell proliferation and mitochondrial dysfunction

**Figure 5:**
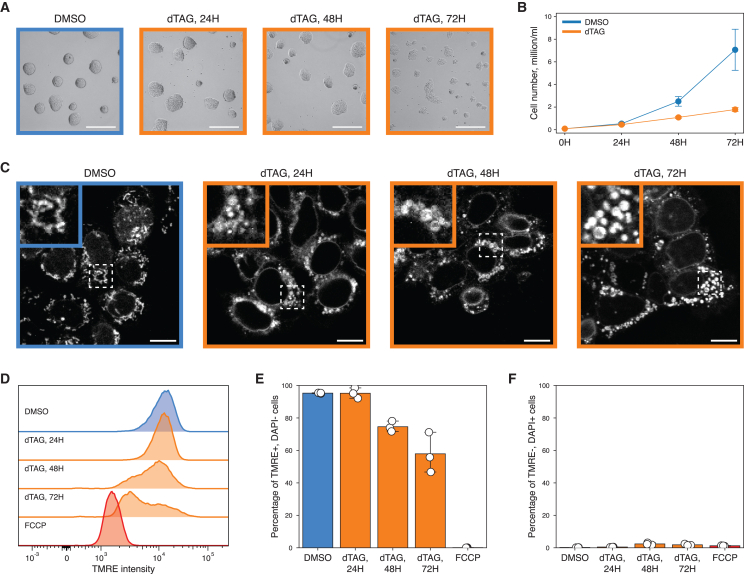
Prolonged loss of ZFP143 impairs cell proliferation and mitochondrial function (A) Bright-field microscopy images showing morphology of DMSO-treated (blue frame) and dTAG-V1-treated (orange frames) mESC colonies. Scale bar: 200 μm. (B) Growth curve showing the total cell number of DMSO-treated (blue line) and dTAG-V1-treated (orange line) cells at different time points. Dots indicate mean values. Error bars indicate standard deviation. (C) Live-cell confocal microscopy images of the MitoTracker Red FM fluorescence in DMSO-treated (blue frame) and dTAG-V1-treated (orange frames) cells. Magnified images of mitochondrial morphology and mitochondrial network for the dash-boxed regions are shown in the upper-left corners. Scale bar: 10 μm. (D) Representative flow cytometry histograms showing TMRE fluorescence distribution in DMSO-treated (blue), dTAG-V1-treated (orange), and FCCP-treated (red) cells. (E) Quantification of TMRE-positive and DAPI-negative cells in DMSO-treated (blue), dTAG-V1-treated (orange), and FCCP-treated (red) cells. Dots represent values for replicates. Error bars indicate 95% confidence interval. (F) Quantification of TMRE-negative and DAPI-positive cells in DMSO-treated (blue), dTAG-V1-treated (orange), and FCCP-treated (red) cells. Dots represent values for replicates. Error bars indicate 95% confidence interval.

To probe the consequences of ZFP143 loss on cell morphology and proliferation, we monitored mESCs over 24, 48, and 72 h of dTAG-V1 treatment. After 24 h of ZFP143 loss, the morphology of the mESCs colonies changes profoundly (Figure 5A). The colonies lose their dome-like structure and tend to flatten out, and this effect becomes particularly clear after 72 h of dTAG-V1 treatment. In addition, cells depleted of ZFP143 showed a clear reduction in cell number, compared with control cells (Figure 5B). This could be a consequence of (1) cell-cycle arrest, (2) slower proliferation rate, or (3) increased cell death. A 5-ethynyl-2′-deoxyuridine (EdU) incorporation assay following ZFP143 depletion did not reveal a shift in the cell-cycle phases, meaning that cells do not undergo cell-cycle arrest (Figures S8A and S8B). However, we observed a decrease in the EdU signal intensity for the S phase cells, suggesting lower EdU incorporation rates (Figure S8C). This observation is consistent with ZFP143-depleted cells spending more time on DNA replication and by extension a slower proliferation rate. Annexin V staining showed a slightly elevated fraction of annexin V-positive cells after 72 h of ZFP143 depletion but not at the earlier time points (Figures S8D–S8F). These findings are in line with changes in the cell cycle and apoptosis gene expression and imply that ZFP143 is essential for maintaining the high proliferation rate of mESCs.

Our transcriptomic and proteomic analyses showed that ZFP143 maintains expression of nuclear-encoded mitochondrial genes. Since mitochondria are involved in the regulation of proliferation, cell cycle, and cell death,^64^^,^^65^ we decided to address the effect of ZFP143 loss on mitochondrial function and visualized mitochondria in living cells using the fluorescent MitoTracker probe.^66^ In control mESCs, we observed elongated mitochondria and mitochondrial networks with high connectivity, while ZFP143-depleted cells present increased amounts of larger, circular mitochondria with fewer interactions (Figure 5C). Such morphological changes have been previously associated with changes in mitochondrial activity,^67^^,^^68^^,^^69^ so we decided to measure the mitochondrial membrane potential using the fluorescent tetramethylrhodamine ethyl ester (TMRE) dye.^70^ Flow cytometry measurements of the TMRE signal demonstrated that ZFP143 depletion resulted in a gradual loss of TMRE intensity (Figures 5D and S8G). The TMRE signal was shifted toward the positive control obtained by treating cells with the mitochondrial uncoupler carbonyl cyanide 4-(trifluoromethoxy)phenylhydrazone (FCCP),^71^ indicating that more depolarized mitochondria were present. Despite the alterations in the mitochondrial proteome and mitochondrial morphology at 24 h post-depletion, we observed a drop in the mitochondrial membrane potential after 48 h of dTAG-V1 treatment, which decreased even further after 72 h (Figures 5E and 5F). Notably, none of the mitochondrial phenotypes described above were observed for the untagged parental mESCs treated with dTAG-V1 (Figure S9). Taken together, the disrupted cell proliferation, altered mitochondrial morphology and loss of mitochondrial membrane potential collectively underline the mitochondrial dysfunction phenotype induced by long-term ZFP143 depletion.

### ZFP143-depleted cells fail to form stem cell-derived embryo models

**Figure 6:**
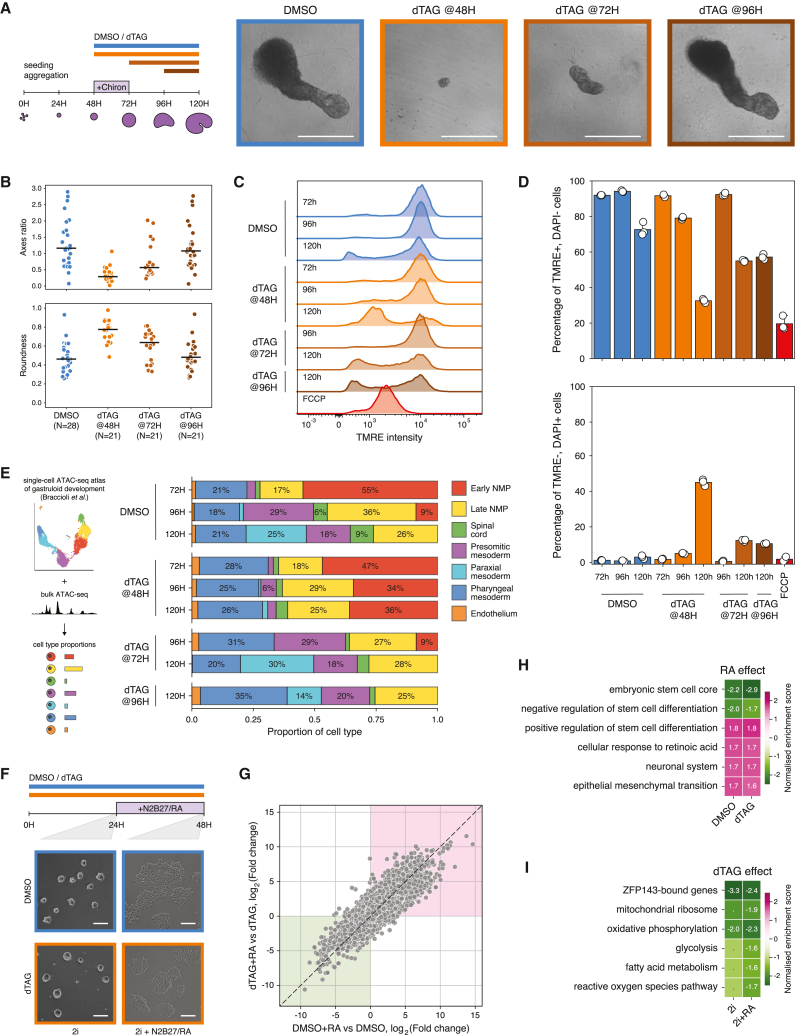
ZFP143-depleted cells fail to develop fully differentiated embryo models despite being able to exit the pluripotent state (A) Schematic of the *in vitro* gastruloids experiment (left). Bright-field microscopy images showing gastruloids treated with DMSO (blue) and dTAG-V1 (orange), imaged at 120 h of development (right). Scale bar: 300 μm. (B) Quantification of axis ratio (top) and roundness (bottom) of gastruloids treated with DMSO (blue) and dTAG-V1 (orange). Black lines indicate the mean values. The number of quantified gastruloids is indicated below. (C) Representative flow cytometry histograms showing TMRE fluorescence distribution in gastruloids treated with DMSO (blue), dTAG-V1 (orange), and FCCP (red) at different stages of development. (D) Quantification of TMRE-positive and DAPI-negative (top) and TMRE-negative and DAPI-positive (bottom) cells in gastruloids treated with DMSO (blue), dTAG-V1 (orange), and FCCP (red) at different stages of development. Dots represent values for replicates. Error bars indicate 95% confidence interval. (E) Quantification of cell-type proportions in gastruloids treated with DMSO or dTAG-V1. The average cell-type proportions of two biological replicates are shown. Colors represent cell-type annotation as referred to in the original study.^74^ (F) Schematic of the retinoic acid differentiation experiment (top). Bright-field microscopy images showing morphology of DMSO-treated (blue frames) and dTAG-V1-treated (orange frames) mESC colonies before (left) and after (right) transfer to N2B27 differentiation media with retinoic acid for 24 h (bottom). Scale bar: 100 μm. (G) Correlation of gene expression changes in DMSO-treated and dTAG-V1-treated cells differentiated with retinoic acid. Each dot represents a single gene, pink and green areas represent concordantly upregulated and downregulated genes, respectively. (H) Gene set enrichment analysis of RNA-seq data, showing the effect of retinoic acid treatment on cells pre-treated with DMSO and dTAG-V1 for 24 h (upregulated gene sets in pink, downregulated gene sets in green). The normalized enrichment score for significant gene sets (FDR < 0.1) is shown. (I) Gene set enrichment analysis of RNA-seq data, showing the effect of dTAG-V1 treatment on cells before (2i) and after transfer to N2B27 differentiation media with retinoic acid (2i+RA) (upregulated gene sets in pink, downregulated gene sets in green). The normalized enrichment score for significant gene sets (FDR < 0.1) is shown.

Studies in zebrafish embryogenesis and mouse hematopoiesis have implicated ZNF143 in development.^24^^,^^26^^,^^38^ However, why ZNF143 is essential for organismal development has remained elusive. To investigate its role in mammalian development, we used an *in vitro* embryo model, called gastruloids, which mimics early stages of post-occipital embryonic development.^72^^,^^73^ Gastruloids are generated by aggregating embryonic stem cells and providing a pulse of the Wnt agonist Chiron, which promotes symmetry breaking and the formation of multiple cell types. We grew gastruloids from the ZFP143-FKBP mESC line and treated them with dTAG-V1 to deplete ZFP143 at different times during development (Figure 6A). ZFP143 depletion at 48 h of development dramatically impaired gastruloid formation, resulting in failure to elongate and the production of small spherical aggregates at 120 h (Figures 6A and 6B). Treatment with dTAG-V1 at 72 h produced a milder phenotype, although these gastruloids were also smaller and less elongated, while treatment at 96 h produced gastruloids morphologically indistinguishable from the control.

To analyze the growth dynamics of gastruloids, we performed time-course imaging at the intermediate time points (Figures S10A and S10B). We found that gastruloids depleted of ZFP143 at 48 h can develop normally up to the 72 h time point, after which they substantially reduce in size and ultimately collapse. Similarly, gastruloids treated with dTAG-V1 at 72 h grow at a similar pace as control gastruloids up to 96 h, after which they shrink. These observations show that in the absence of ZFP143, gastruloids continue to grow normally for 24 h, after which they begin to shrink and even collapse.

To check whether the growth defect is accompanied by mitochondrial depolarization in gastruloids, we measured the mitochondrial membrane potential at different stages of gastruloid development (Figure 6C). In gastruloids depleted of ZFP143 at 48 and 72 h, we observed an increase in cells with depolarized mitochondria at 96 and 120 h of development, respectively (Figure 6D). Similar to mESCs, the effects of ZFP143 loss on mitochondrial function were manifested by 48 h of dTAG-V1 treatment. Interestingly, in dTAG-V1-treated gastruloids, but not in mESCs, the cells that lose TMRE signal also gain DAPI signal, indicating ongoing cell death,^75^ consistent with their growth dynamics (Figures 6D and S10A). Our data reveal that mESCs and gastruloids show analogous phenotypes following ZFP143 depletion and suggest that the proliferation defect in gastruloids may be partly explained by the mitochondrial dysfunction.

We next sought to determine the effect of ZFP143 loss on the cell-type composition of gastruloids. To estimate cell-type proportions, we performed bulk chromatin accessibility measurements by assay for transposase-accessible chromatin with sequencing (ATAC-seq) and deconvoluted the signal using a previously generated time-resolved single-cell chromatin accessibility atlas of gastruloid development.^74^ Deconvolution analysis shows that at 72 h of development, control gastruloids are mainly composed of progenitor cells, resembling the epiblast-like state^74^^,^^76^ (Figure 6E). This progenitor population is dramatically reduced by 96 h, when cells exit the pluripotent state, and give rise to more differentiated cell types. However, in gastruloids depleted of ZFP143 at 48 h of development, progenitor cells still make up the largest cell population at 96 h, whereas in gastruloids depleted of ZFP143 at 72 and 96 h, we observed the same cell-type proportions as in control gastruloids (Figure 6E).

We then aimed to understand whether ZFP143 is required to maintain the differentiation capacity of stem cells by facilitating the exit from the pluripotent state. To this end, we asked whether ZFP143-depleted mESCs could differentiate in 2D culture upon treatment with retinoic acid (RA), the widely used signaling molecule.^77^^,^^78^ We first pre-treated mESCs with either dTAG-V1 or DMSO for 24 h, and we then induced differentiation by adding RA for the next 24 h while keeping the cells in dTAG-V1 or DMSO, respectively. Treatment of cells with RA had no further effect on the proliferation rates of control and ZFP143-depleted mESCs (Figure S11A), and both were able to acquire neural-like morphology, indicating successful RA-induced neuronal differentiation (Figure 6F).

To determine whether differentiation with RA induces similar transcriptomic changes in control and ZFP143-depleted cells, we performed RNA-seq before and after RA treatment. Principal-component analysis showed that RA treatment induces similar changes in control and dTAG-V1-treated mESCs, accounting for 83% of the variance between samples, while the difference induced by ZFP143 depletion represents only 14% of the variance (Figure S11B). Differential expression analysis in RA-treated control and ZFP143-depleted cells revealed that genes in both conditions have a high degree of overlap and show concordant expression changes (Figures 6J, S11C, and S11D). Furthermore, GSEA showed that both control and ZFP143-depleted cells show a reduced expression of stem cell-related genes and upregulate genes involved in RA response, neural lineage differentiation, and epithelial-to-mesenchymal transition (Figure 6H). Examining the effects of ZFP143 depletion in cells before and after RA treatment revealed downregulation of nuclear-encoded mitochondrial genes involved in energy metabolism and redox homeostasis (Figure 6I). This confirms that ZFP143 is not directly required for stem cell differentiation in monoculture but maintains a cellular state that drives proper organismal development in a multicellular context.

## Discussion

Numerous studies have suggested that ZNF143 functions as a general looping factor, cooperating with CTCF in establishing enhancer-promoter interactions. Here, we were unable to identify a role for ZFP143 in chromatin looping by examining the interaction landscape using Hi-C and 4C-seq following acute loss of ZFP143. An accompanying study^79^ found that even in nucleosome-resolution chromosome conformation maps generated with Micro-C, no consistent changes in interactions could be observed upon acute ZNF143 loss. It should be noted that previous studies suggesting a role for ZNF143 in chromatin looping show only mild effect sizes, particularly when compared with depletion of well-established regulators of chromatin organization such as CTCF,^80^^,^^81^ cohesin,^82^^,^^83^ WAPL,^84^^,^^85^ or NIPBL.^86^^,^^87^ Furthermore, the reported changes were detected in a non-acute ZNF143 depletion setting,^38^^,^^39^^,^^46^^,^^47^ which means that these observations could also be an indirect consequence of prolonged ZNF143 loss.

Systematic re-analysis of the ZNF143 ChIP-seq data revealed that the Proteintech polyclonal anti-ZNF143 antibody recognizes CTCF in addition to ZNF143. Our ChIP-seq data with the Proteintech antibody in the absence of ZNF143 confirmed antibody cross-reactivity with CTCF. To our knowledge, the only studies that reported an overlap between ZNF143 and CTCF utilized the data generated with this antibody.^30^^,^^31^^,^^33^^,^^34^^,^^35^^,^^39^ Re-use of the same data led to the observed enrichment of ZNF143 at chromatin loop anchors,^35^^,^^36^^,^^40^ causing misassociation of ZNF143 with chromatin looping. This pitfall questions the use of polyclonal antibodies for ChIP-seq experiments^88^ and emphasizes the need for vigilance in the interpretation of data generated with these antibodies.^89^ Taken together, our results rule out a general looping factor role for ZNF143, which has important implications for 3D genome organization and for the interpretation of gene regulation mediated by ZNF143.

By combining transcriptional changes induced by acute ZFP143 loss with ZFP143 chromatin binding, we were able to confidently identify direct targets of ZFP143. We found that loss of ZFP143 leads to downregulation of hundreds of genes, the vast majority of which have ZFP143 binding at the promoter. Moreover, we observed a profound, yet delayed, correlation between transcription and protein levels of these genes, which may indicate a global housekeeping function for ZFP143.^90^^,^^91^^,^^92^^,^^93^^,^^94^ We found that many of ZFP143 target genes are nuclear-encoded mitochondrial proteins, including components of mitochondrial ribosome and oxidative phosphorylation complexes. While post-transcriptional regulation of mitochondrial proteins is well studied,^95^^,^^96^^,^^97^ little is known about the transcriptional regulation of these genes, and our dataset can serve as a useful starting point for future research on this topic.

We found that most ZFP143-bound genes show no change in their expression following depletion, indicating that ZFP143 is not the primary activator at many binding sites. The role of ZFP143 at these regions is an interesting question for future research, given that they partially comprise a conserved set of ZNF143-bound promoters between cell types and species. One explanation may be that TFs, such as HCFC1, THAP11, and others, which overlap with ZFP143 binding,^12^^,^^19^^,^^98^^,^^99^^,^^100^ cooperate or compete with ZFP143 at these promoters and may take over the regulatory function in its absence. Another possibility is that ZFP143 is required for activation at some of these sites in different cell types thus posing its role as a cell-type-specific TF. Lastly, ZFP143 binding sites were also found to overlap with ICN1, the active form of the transmembrane receptor NOTCH1,^12^^,^^101^ suggesting it may be involved in signaling responses. Considering the auto-regulatory loop regulating ZFP143^102^ and its long chromatin residence time,^79^ we speculate that ZFP143 may be required for rapid transcriptional responses to accommodate specific metabolic conditions and developmental cues.

We established that depletion of ZFP143 for more than 24 h resulted in reduced proliferation and mitochondrial dysfunction. The observed phenotype is similar to the direct effects on mitochondrial function by inhibiting mitochondrial translation,^103^^,^^104^^,^^105^ affecting oxidative phosphorylation complexes,^103^^,^^105^^,^^106^^,^^107^ or shifting the balance between nuclear- and mitochondrial-encoded mitochondrial proteins.^108^^,^^109^ ZNF143 loss has previously been associated with a downregulation of cell-cycle genes^12^^,^^17^^,^^19^; however, these experiments were done in a non-acute setting. The fact that it takes more than 24 h after depletion to see the first effects on cell proliferation suggests that ZFP143 is not directly involved in the regulation of the cell cycle. Rather, the proliferation defect is likely to be a consequence of the mitochondrial phenotype, which may result in reduced energy production.^105^^,^^110^^,^^111^^,^^112^^,^^113^ In this case, ZFP143 contributes positively to proliferation by regulating genes involved in energy homeostasis.

Our findings are consistent with existing literature suggesting that ZNF143 is crucial for development. Although our results do not directly implicate ZFP143 in regulating pluripotency genes,^23^ we observed an upregulation of differentiation genes after 24 h of ZFP143 depletion. However, ZFP143 depletion did not lead to rapid changes in morphology and differentiation, as reported for *bona fide* pluripotency TFs OCT4 or SOX2.^114^^,^^115^ Paradoxically, loss of ZFP143 in the early stages of gastruloid development results in differentiation failure, as cells in these gastruloids do not progress beyond the progenitor state. Since ZFP143-deficient cells can exit the pluripotent state upon RA treatment, it seems plausible that mitochondrial homeostasis maintained by ZFP143 is critical for gastruloid development, particularly at the early stages.^75^^,^^116^ The absence of this effect at later treatment times may be explained by the fact that the gastruloids have already undergone the necessary molecular and metabolic changes.^117^^,^^118^^,^^119^ Our results indicate that in a multicellular embryo model, as opposed to a cell line model, differentiation requires ZFP143.

Our data place ZNF143 at the center of a gene regulatory network that controls mitochondrial function. By maintaining gene expression of a subset of nuclear-encoded mitochondrial genes, ZNF143 plays a critical role in maintaining cellular homeostasis, which may explain its previously identified roles in a variety of biological processes in normal and malignant conditions. We expect that our study will bring the focus back to the function of ZNF143 as an essential transcriptional activator, as it was originally described.

### Limitations of the study

In our study, we have ruled out a general looping function for ZNF143. However, the Hi-C data have limited resolution, and therefore we cannot exclude a role for ZNF143 in looping at a small number of specific sites. In addition, ZNF143 binds and activates many genes besides nuclear-encoded mitochondrial genes. The contribution of these genes and their products to the observed phenotypes and the overlap in their regulation by other TFs should be further investigated, particularly as our results show that ZNF143 is critical for multicellular development. While our work used gastruloids, which capture important features of post-occipital development, future experiments are needed to investigate the role of ZNF143 in more complex developmental models.

## Resource availability

### Lead contact

Further information and requests for resources and reagents should be directed to and will be fulfilled by the lead contact, Elzo de Wit (e.d.wit@nki.nl).

### Materials availability

Plasmids generated in this study have been deposited to Addgene (ZFP143-FKBP-donor and ZFP143-sgRNA, Addgene #212705 and #212706). ZFP143-FKBP mESC cell line generated in this study is available upon reasonable request from the lead contact, Elzo de Wit (e.d.wit@nki.nl).

### Data and code availability

● ChIP-seq, Hi-C, 4C-seq, TT-seq, and RNA-seq data generated for ZFP143-FKBP mESCs have been deposited at the GEO database (GSE260914) and are publicly available as of the date of publication. ChIP-seq data generated for ZNF143-FKBP HEK293T cells have been deposited at the GEO database (GSE271837) and are publicly available as of the date of publication. Proteomics data have been deposited at the PRIDE database (PXD049480) and are publicly available as of the date of publication. Previously published ChIP-seq datasets used in this study are listed in Table S2 and can be accessed from the GEO database. Original western blots, microscopy images, flow cytometry measurements, cell count data, differential expression and GSEAs results, and gastruloids quantifications have been deposited at the Mendeley Data (https://doi.org/10.17632/vd5473hvbr) and are publicly available as of the date of publication. All accession numbers are listed in the key resources table.
● All original code has been deposited at Zenodo (https://doi.org/10.5281/zenodo.14168879) and is publicly available as of the date of publication.
● Any additional information required to reanalyze the data reported in this paper is available from the lead contact upon request.

## Acknowledgments

We thank the NKI Flow Cytometry Facility, NKI Genomics Core Facility, NKI Proteomics Facility, NKI BioImaging Facility, and NKI Research High Performance Computing Facility. We thank Anders Hansen and Domenic Narducci for the exchange of unpublished results. We thank Koen Flach for help with cell line generation. We thank Moreno Martinović and Teun van den Brand for help with 4C-seq and ATAC-seq data analysis. We thank William Faller for fruitful discussions. We thank members of our lab for critically reading the manuscript. Research at the Netherlands Cancer Institute is supported by an institutional grant of the Dutch Cancer Society and of the Dutch Ministry of Health, Welfare and Sport. Work in the de Wit lab is supported by the Dutch Research Council (016.161.316, Vidi; VI.C.222.049, Vici) and the European Research Council (865459, “FuncDis3D”). The NKI Proteomics Facility is supported by the Dutch Research Council X-omics Initiative.

## Author contributions

M.D.M., M.M., and E.d.W. conceived and designed the study. M.M. engineered and characterized ZFP143-FKBP mESC line. M.M., N.A.S., and H.T. prepared sequencing libraries. M.M., N.A.S., and H.T. performed functional and validation experiments. M.M., N.A.S., and L.B. performed gastruloid experiments. J.D. and K.M.S. performed ChIP-seq experiments in HEK293T cells under supervision of M.J.G. M.D.M. analyzed sequencing, mass spectrometry, flow cytometry, microscopy, and publicly available data. E.d.W. supervised the study. M.D.M., M.M., and E.d.W. wrote the manuscript with input from all authors. All authors read and approved the manuscript.

## Declaration of interests

The authors declare no competing interests.

## STAR★Methods

### Key resources table

**Table:** 

REAGENT or RESOURCE	SOURCE	IDENTIFIER
**Antibodies**		

Anti-ZNF143	Proteintech	Cat. #16618-1-AP; RRID:AB_2218324
Anti-HA tag	Abcam	Cat. #ab9110; RRID:AB_307019
Anti-ɑ-Tubulin	Sigma-Aldrich	Cat. #T5168; RRID:AB_477579
Anti-CTCF	Merck Millipore	Cat. #07-729; RRID:AB_441965
Anti-CTCF	Cell Signalling	Cat. #3418S; RRID:AB_2086791

**Bacterial and virus strains**		

5-alpha competent bacteria	NEB	Cat. #C2987

**Chemicals, peptides, and recombinant proteins**		

dTAG-V1	Torcis	Cat. #6914
AMPure XP beads	Beckman Coulter	Cat. #A63881
Dynabeads Protein-A	Invitrogen	Cat. #10002D
Dynabeads Protein-G	Invitrogen	Cat. #10003D/10004D
NEBuilder HiFi DNA Assembly Master Mix	NEB	Cat. #E2621L
DNase I recombinant, RNase-free	Roche	Cat. #04716728001
All-trans Retinoic acid	Sigma-Aldrich	Cat. #R2625
Neurobasal medium	Gibco	Cat. #21103-049
DMEM/F12 medium	Gibco	Cat. #11320-033
DMEM medium	Gibco	Cat. #11965092
Neurobasal medium, no phenol red	Gibco	Cat. #12348-017
DMEM/F-12, no phenol red	Gibco	Cat. #21041-025
N2	Gibco	Cat. #17504-044
B27	Gibco	Cat. #17502-048
LIF	Sigma-Aldrich	Cat. #ESG1107
CHIR-99021	MedChemExpress	Cat. #HY-10182
PD0325901	MedChemExpress	Cat. #HY-10254
4-Thiouridine	Sigma-Aldrich	Cat. #T4509
Monothioglycerol	Sigma-Aldrich	Cat. #M6145-25ML
Clarity Western ECL Substrate	Bio-Rad	Cat. #1705061
DAPI	Sigma-Aldrich	Cat. #MBD0015
TMRE	Thermo Scientific	Cat. #T669
Annexin V APC	BioLegend	Cat. #640920
MitoTracker Red FM	Thermo Scientific	Cat. #M22425
Biolaminin	BioLamina	Cat. #LN511

**Critical commercial assays**		

Click-iT EdU Alexa Fluor 647 Flow Cytometry Assay Kit	Invitrogen	Cat. #C10424
MinElute PCR Purification Kit	Qiagen	Cat. #28004/28006
RNeasy Mini Kit	Qiagen	Cat. #74104
μMacs Streptavidin Kit	Miltenyi	Cat. ##130-074-101
KAPA RNA HyperPrep Kit	Roche	Cat. #KK8540
KAPA HTP Library Preparation Kit	Roche	Cat. #KR0426
KAPA Dual-Indexed Adapter Kit	Roche	Cat. #KR1736
TruSeq Stranded mRNA Kit	Illumina	Cat. #20020594
TruSeq RNA Single Indexes	Illumina	Cat. #20020492
KAPA HiFi HotStart ReadyMix PCR Kit	Roche	Cat. #KK2602
PureLink HiPure Plasmid Filter Maxiprep Kit	Invitrogen	Cat. # K210016
Mouse Embryonic Stem Cell Nucleofector Kit	Lonza	Cat. #VPH-1001
MycoAlert Mycoplasma Detection Kit	Lonza	Cat. #LT07-318
Pierce Bradford Protein Assay Kit	Thermo Scientific	Cat. #23200
NEBNext Ultra II DNA Library Prep Kit	NEB	Cat. #E7103S/L
NEBNext Multiplex Oligos for Illumina	NEB	Cat. #E7335S/L, #E7500S/L

**Deposited data**		

ChIP-seq, TT-seq, Hi-C, 4C-seq, RNA-seq, and ATAC-seq data in mESCs	This study	GEO: GSE260914
ChIP-seq data in HEK293T cells	This study	GEO: GSE271837
Proteomics data	This study	PRIDE: PXD049480
Western blots, microscopy images, flow cytometry measurements, cell count data, and ATAC-seq quantifications	This study	Mendeley Data: 10.17632/vd5473hvbr
Publicly available ChIP-seq data, see Table S2	N/A	N/A

**Experimental models: Cell lines**		

E14TG2a mESCs	ATCC	Cat. #CRL-1821
ZFP143-FKBP E14TG2a mESCs	This study	N/A

**Oligonucleotides**		

gRNA, primers for genotyping, 4C-seq, and ATAC-seq, see Table S1	This study	N/A

**Recombinant DNA**		

ZFP143-FKBP-donor vector	This study	Addgene #212705
ZFP143-sgRNA vector	This study	Addgene #212706

**Software and algorithms**		

FlowJo v10.9.0	Treestar	RRID: SCR_008520
ImageJ v2.9.0	NIH	RRID: SCR_003070
bwa v0.7.17-r1188	Li and Durbin^120^	RRID: SCR_010910
SAMtools v1.12	Li et al.^121^	RRID: SCR_002105
Picard v2.25.6	Broad Institute	RRID: SCR_006525
BEDTools v2.27.1	Quinlan and Hall^122^	RRID: SCR_006646
deepTools v3.4.2	Ramírez et al.^123^	RRID: SCR_016366
pyGenomeTracks v3.8	Lopez-Delisle et al.^124^	RRID:SCR_025312
MACS2 v2.2.6	Zhang et al.^125^	RRID: SCR_013291
meme v5.4.1	Bailey et al.^126^	RRID: SCR_001783
ica	Nathaniel Helwig	https://CRAN.R-project.org/package=ica
motifmatchr	Schep^127^	https://bioconductor.org/packages/motifmatchr/
regioneR	Get et al.^128^	https://bioconductor.org/packages/regioneR/
JASPAR2020	Baranasic^129^	https://bioconductor.org/packages/JASPAR2020/
g:Profiler	Raudvere et al.^130^	RRID: SCR_006809
liftOver	UCSC	RRID: SCR_018160
biomaRt	Durinck et al.^131^	RRID: SCR_019214
bioframe v0.3.3	Open2C^132^	https://github.com/open2c/bioframe
distiller-nf	Open2C	https://github.com/open2c/distiller-nf
pairtools v0.3.0	Open2C^133^	RRID: SCR_023038
cooler v0.8.11	Abdennur and Mirny^134^	RRID: SCR_024194
cooltools v0.5.1	Open2C^135^	https://github.com/open2c/cooltools
coolpup.py v0.9.5	Flyamer et al.^136^	https://github.com/open2c/coolpuppy
STAR v2.7.9a	Dobin et al.^137^	RRID: SCR_004463
HTSeq v0.13.5	Anders et al.^138^	RRID: SCR_005514
DESeq2 v1.30.1	Love et al.^139^	RRID: SCR_015687
GSEA v4.3.2	Subramanian et al.^140^	RRID: SCR_003199
DIA-NN v1.8	Demichev et al.^141^	RRID: SCR_022865
Perseus v2.0.10.0	Tyanova et al.^142^	RRID: SCR_015753

**Other**		

Trans-Blot turbo transfer system	Bio-Rad	Cat. #1704275
μ-Slide 8-well microscopy slide	ibidi	Cat. #80826
HC PL APO 63x/1,40 OIL CS2 objective	Leica	Cat. #11506350

### Experimental model and study participant details

#### Cell lines

Mouse Embryonic Stem Cells E14Tg2A (129/Ola) cell lines were cultured on 0.1% gelatin-coated plates in serum-free DMEM/F12 (Gibco) and Neurobasal (Gibco) medium (1:1) supplemented with N-2 (Gibco), B-27 (Gibco), BSA (0.05%, Gibco), 10 × 4 U of Leukaemia Inhibitory Factor (LIF) (Millipore), MEK inhibitor PD0325901 (1 μM, Selleckchem), GSK3-β inhibitor CHIR99021 (3 μM, Cayman Chemical) and 1-Thioglycerol (1.5 × 10^−4^ M, Sigma-Aldrich). The cell lines were passaged every 2 days in daily culture. For treatment using small molecule degraders, cells were counted and seeded as normal. The day after seeding DMSO or dTAG-V1 at a final concentration of 500nM (in DMSO) were added to cell medium. Molecules and media were refreshed every 24h if needed. Cells were tested for Mycoplasma routinely (Lonza).

Human Embryonic Kidney 293T ZNF143-FKBP12^F36V^ cells^58^ were cultured in DMEM (Gibco) supplemented with 10% fetal bovine serum (FBS), Penicillin/Streptomycin, and 5% glutamine at 37°C with 5% CO2.

#### Gene targeting

For the knockin of the FKBP sequence at the genes of interest, previously described approach^143^ and plasmids to recognize the endogenous Zfp143 locus were used (ZFP143-FKBP-donor and ZFP143-sgRNA, Addgene #212705 and #212706). The DNA sequence for the knockin was designed to include the FKBP-2xHA-P2A-GFP cassette in between two homology arms for surrounding the STOP codon of *Znf143* gene and ordered from Twist Bioscience. Plasmids were amplified in competent bacteria and prepared for nucleofection following Nucleofector kit protocol (Lonza). The cells were transfected with the plasmids containing the gRNA sequence and the donor plasmid designed to include the FKBP-2xHA-P2A-GFP in between two homology arms for the gene of interest. After transfection, cells were grown in 2i medium. After expansion, single cells with a positive GFP signal were sorted in 96-well plates and manually picked for genotyping using PCR and western blotting. Homozygous clones responding to dTAG-V1 were used for experiments. Primers and gRNA sequences are listed in Table S1.

### Method details

#### Cell count

For cell count, 4x10^4^ ZFP143-FKBP cells were seeded. The following day the cells were counted to get 0 hour point and then DMSO or dTAG-V1 at a final concentration of 500 nM were added directly into the medium. Medium supplemented with treatment molecules was refreshed daily. For counting after 24, 48, and 72 hours or treatment, cells were harvested by trypsinization with TVP1X, gently dissociated into single cells and counted by an automated cell counter (Bio-Rad). For cell count during retinoic acid differentiation, the same procedure was followed, replacing the media with N2B27 medium supplemented with RA after 24 hours of pre-treatment with DMSO or dTAG-V1. All cell count experiments were performed in technical triplicates and repeated for two biological replicates.

#### Western blotting

Whole cell lysate was collected by adding RIPA Lysis buffer (150 mM NaCl, 1% NP-40, 0.5% Sodium Deoxycholate, 0.1%SDS, 25mM Tris (pH 7.4)) to the cells for 1 hour. Proteins were quantified using Bradford assays. 40μg of proteins were loaded and separated on an in-house prepared 10% SDS-PAGE gel. After transfer on a PVDF pre-activated membrane using the Trans-Blot turbo transfer system (Bio-Rad), membrane proteins were blocked with 5% milk. Blots were incubated with primary antibodies (HA, ab9110, Abcam, 1:2000; ZNF143, 16618-1-AP, Proteintech 1:1000; ɑ-Tubulin, T5168, Sigma-Aldrich, 1:5000) for 1 hour at room temperature. Species-specific secondary antibodies were incubated for 1 hour at room temperature. After incubation, blots were washed at least three times in 0.1% Tween-20 in TBS. Proteins were detected using the Clarity Western ECL Substrate (Bio-Rad) with ChemiDoc MP Imaging system (Bio-Rad).

#### ChIP-seq

For chromatin preparation, mESCs cells were grown and treated with 500 nM dTAG-V1 or DMSO for ZFP143 depletion. Cells were harvested and collected to have 1x10^6^ cells/ml. For CTCF ChIP-seq, 10% of HCT116 cells were added to mESC cells and used as an internal reference. Chromatin was cross-linked using 1% FA final concentration at RT for 10 min. Glycine 2.0 M was added directly after to quench the cross-linking reaction. Fixed cells were lysed and chromatin was sheared on a Bioruptor Plus sonication instrument (Diagenode) to achieve chromatin fragments between 200 and 500 bp. For the IP, antibodies were coupled overnight with Protein G Dynabeads (Thermo Scientific). The following day, antibodies-coupled beads were washed and the sheared chromatin was added. Antibodies against CTCF (07-729, Merck Millipore, 5 μl per ChIP) and HA (ab9110, Abcam, 10 μl per ChIP) were used. After incubation of the chromatin and antibody-beads overnight at 4°C, the chromatin fragments captured on beads were de-crosslinked from proteins and DNA fragments were released. DNA was purified using the MinElute PCR purification kit (Qiagen). The resulting DNA was used for preparation of the sequencing library, following the KAPA HTP Library Preparation kit (Roche). Libraries were sequenced on Illumina NextSeq 550 generating paired-end reads.

HEK293T ZNF143-FKBP12^F36V^ cells were grown to approximately 80-90% confluence in 10 cm dishes. The cells were treated with 0.5 μM dTAGV-1 for 30 minutes, or no treatment for control. Formaldehyde (final concentration 1%, Electron Microscopy Sciences) was added directly to the plates containing 10 mL of media and incubated for 10 minutes at 37°C in the incubator to fix the cells. The reaction was then quenched by adding 0.125M glycine (final concentration) to the plates, swirling, and incubating for an additional 10 minutes at 37°C. The plates were washed once with 10 mL and then with 5 mL of ice-cold PBS containing protease inhibitors (cOmplete Roche, Sigma-Aldrich). Subsequently, the cells were scraped into 5 mL of ice-cold PBS containing protease inhibitors and transferred to a 15 mL tube. The process was repeated to collect all the cells, and the pellet was transferred to a 1.5 mL tube and stored at -80°C after snap freezing in liquid nitrogen. For ChIP, the frozen pellets were thawed on ice for 10 minutes. The cells were then lysed by resuspending in 1 mL of cold sonication buffer per 1 x 108 cells (0.5% SDS, 0.01M EDTA, 50mM Tris-HCl pH 8.0, and 1X protease inhibitors). The lysate was divided to obtain approximately 200 to 300 μL per tube and sonicated for 15 sec ON, 15 sec OFF, for 30 minutes at 25% amplitude using a Qsonica sonicator. The samples were then spun at ∼17000x g in a mini centrifuge for 10 minutes at 4°C, and the supernatant was collected. The supernatant was diluted (at least 5 times) to achieve a concentration of one million cells per 200 μL lysate to reduce SDS concentration with ChIP dilution buffer containing protease inhibitors (0.01% SDS, 1.2mM EDTA, 16.68 mM Tris-HCl pH 8.0, 167mM NaCl, 1.1% Triton X-100, and 1X protease inhibitors). Four million cell equivalents of cell lysate were aliquoted, and appropriate amounts of antibody were added (4 μL of anti-CTCF antibody (3418S, Cell Signalling, 91ng/μl) or 18μL of anti-ZNF143 antibody (16618-1-AP, Proteintech, 500 ng/μl)). The samples were incubated overnight with rotation at 4°C. The immune complex was collected by incubating with 40 μL Protein A + Protein G beads (Dynabeads Protein A and Protein G, Invitrogen) on a rotisserie at 4°C for 90 minutes. The beads were washed once each with low salt immune complex buffer (0.1% SDS, 1% Triton x-100, 2mM EDTA, 150mM NaCl, 20mM Tris-HCl pH 8.0), high salt immune complex buffer (0.1% SDS, 1% Triton x-100, 2mM EDTA, 500mM NaCl, 20mM Tris-HCl pH8.0), LiCl immune complex buffer (0.25M LiCl, 1% NP-40, 1% deoxycholate, 1mM EDTA, 10mM Tris-HCl pH8.0), and then with 1x TE (10mM Tris-HCl, 1mM EDTA pH8.0), all containing 1x protease inhibitors. To elute the immunoprecipitated complexes, 150 μL of elution buffer (10mM Tris-HCl pH 8.0, 300mM NaCl, 55mM EDTA, 0.5% SDS) was added, followed by 1 μL RNAse A, and incubated at 37°C for 10 minutes. This was followed by the addition of 5 μL Proteinase K and overnight incubation at 65°C. The DNA was purified after removing the beads with a MinElute PCR purification kit (Qiagen), and eluted DNA in a 52 μL elution buffer provided in the kit. ChIP-seq libraries were prepared using the NEBNext Ultra II DNA Library Prep Kit (NEB) and NEBNext Multiplex Oligos for Illumina (NEB) index primers, following the manufacturer's protocols. Libraries were assessed on a TapeStation and Qubit and sequenced on Illumina NextSeq 500 generating paired-end reads.

#### Hi-C

Hi-C data were generated as previously described^40^ with minor modifications.^84^ For each template, 10 million cells were harvested and crosslinked using 2% formaldehyde. Crosslinked DNA was digested in the nucleus using MboI (NEB), and biotinylated nucleotides were incorporated at the restriction overhangs and joined by blunt-end ligation. The ligated DNA was enriched in a streptavidin pull-down. Hi-C libraries were prepared using a standard end-repair and A-tailing method and sequenced on Illumina MiSeq and NextSeq 550 generating paired-end reads.

#### 4C-seq

4C was performed for DMSO and 500 nM dTAG-V1 treated ZFP143-FKBP cells as previously described^55^^,^^144^ using a two-step PCR method for indexing.^84^ 10 million cells were used for each time-point. MboI (NEB) was used as the first restriction enzyme and Csp6I (NEB) as the second restriction enzyme. Viewpoint-specific primers are listed in Table S1. 4C-seq was performed in two biological replicates. Libraries were sequenced on Illumina NextSeq 550 generating paired-end reads.

#### TT_chem_-seq

Libraries for TT_chem_-seq were prepared following a published protocol.^51^ ZFP143-FKBP cells were seeded and the day after treated with 500 nM of DMSO or dTAG-V1 for 2, 6, and 24 hours. Cells were labelled with 2 mM of the uridine analog 4-thiouridine (Sigma-Aldrich) for 10 min. Total RNA was isolated and fragmented. The 4sU-biotin labelled RNA was enriched using μMacs Streptavidin Kit (Miltenyi). Libraries were prepared using KAPA RNA HyperPrep and KAPA Dual-Indexed Adapter kits (Roche) using dual indexing adapters. TT_chem_-seq was performed in two biological replicates. Libraries were sequenced on Illumina NextSeq 550 generating paired-end reads.

#### RNA-seq

For RNA-seq, cells were harvested, kept in an RLT buffer stored at -80°C until purification or were directly used for RNA purification. RNA was first isolated using standard RNA isolation procedure from Qiagen RNeasy Mini kit (Qiagen), including a DNaseI treatment. The resulting RNA was used directly for library preparation following the TruSeq Stranded mRNA kit (Illumina) with TruSeq RNA Single Indexes set A (Illumina). RNA-seq was performed in two biological replicates. Libraries were sequenced on Illumina NextSeq 550 generating paired-end reads.

#### Quantitative mass spectrometry

For quantitative measure of protein abundance, ZFP143-FKBP and untagged parental mESCs were expanded and treated with either DMSO or 500 nM dTAG-V1 for 2, 6 and 24 hours. Cells were harvested and 30 million cells per condition were centrifuged at 500g for 5 minutes, washed with PBS and pellets were snap frozen. All samples were prepared in triplicate.

For protein digestion, frozen pellets were lysed in boiling Guanidine (GuHCl) lysis buffer as previously described.^145^ Protein concentration was determined with a Pierce Coomassie (Bradford) Protein Assay kit (Thermo Scientific), according to the manufacturer’s instructions. After dilution to 2M GuHCl, aliquots corresponding to 200μg of protein were digested twice (4h and overnight) with trypsin (Sigma-Aldrich) at 37°C, enzyme/substrate ratio 1:75. Digestion was quenched by the addition of FA (final concentration 5%), after which the peptides were desalted on a Sep-Pak C18 cartridge (Waters) and dried in a vacuum centrifuge. Prior to mass spectrometry analysis, the peptides were reconstituted in 2% formic acid. Peptide mixtures were analysed by nano LC-MS/MS on an Orbitrap Exploris 480 Mass Spectrometer equipped with an EASY-nLC 1200 system (Thermo Scientific). Samples were directly loaded onto the ReproSil-Pur 120 C18-AQ analytical column (2.4 μm, 75 μm × 500 mm, packed in-house). Solvent A was 0.1% formic acid/water and solvent B was 0.1% formic acid/80% acetonitrile. Samples were eluted from the analytical column at a constant flow of 250 nl/min in a 90-min gradient, containing a 78-min linear increase from 6% to 30% solvent B, followed by a 12-min wash at 90% solvent B.

#### Flow cytometry

##### Cell cycle assay

Cell cycle analysis was performed following the protocol of the Click-iT EdU Alexa Fluor 647 Flow Cytometry Assay kit (Invitrogen). Briefly, cells were counted, seeded, and treated with either DMSO or dTAG-V1 the following day. For labelling, 10 μM EdU was added to cells, for 1.5 h and incubated at 37°C. After this time cells were harvested, fixed, and permeabilized using a saponin-based permeabilization and wash reagents provided from the manufacturer. To detect EdU, cells were incubated with Click-iT reaction cocktail for 30 min. For DNA content measure, RNA was removed adding 10 μg/ml Ribonuclease A and DAPI (1:1000) was added to stain for DNA content. EdU and DAPI fluorescent signals were quantified on a BD LSRFortessa analyzer (BD Biosciences) with the R(C) 670/30 and V(F) 450/50 laser settings. Cell count assay was performed in technical triplicates and repeated for one biological replicate. Subsequent analysis was done with FlowJo v10.9.0 software. Single cells were gated, and G1, S and G2 cells were separated and quantified using the indicated gates.

##### Apoptosis assay

Apoptosis detection was performed by using Annexin V APC (BioLegend). After harvesting, the cells were resuspended in 150 μl binding buffer (0.1 M HEPES pH 7.4, 1.4 M NaCl, 25 mM CaCl2) and spinned down. The supernatant was discarded and 300 μl of Annexin V Binding Buffer, 3 μl of Annexin V APC (stock concentration 8μg/ml) and 3 μl DAPI (stock concentration 70μg/ml) were added to the cells. The cells were incubated for 20 minutes at room temperature and Annexin V and DAPI fluorescent signals were then quantified on a BD FACSAria Fusion analyzer (BD Biosciences) with the R(C) 670/30 and V(F) 450/50 laser settings. Apoptosis assay was performed in technical triplicates and repeated for two biological replicates. Subsequent analysis was done with FlowJo v10.9.0 software. All single cells were gated, and healthy, early apoptotic, and late apoptotic cells were then separated and quantified using the indicated gates.

##### Mitochondrial membrane potential assay

Mitochondrial membrane potential was measured by using TMRE (Thermo Scientific). Cells were trypsinized, washed with PBS and made single cells in suspension. As a control, 20 μM carbonyl cyanide 4-(trifluoromethoxy)phenylhydrazone (FCCP) was added to one of the samples and incubated for 30 minutes. Next, cells were incubated with 100 nM TMRE (Thermo Scientific) for 30 minutes. DAPI at working concentration 1μg/ml was added to the cells 15 mins prior to sorting. TMRE and DAPI signals were detected on a BD LSRFortessa analyzer (BD Biosciences) with the YG(E) 585/15 and V(F) 450/50 laser settings. Mitochondrial membrane potential assay was performed in technical triplicates and repeated for one biological replicate. Subsequent analysis was done with FlowJo v10.9.0 software. Single cells were gated, and TMRE-positive cells were then separated and quantified using the indicated gates.

#### Live-cell imaging of mitochondria

ZFP143-FKBP and untagged parental E14 mESC were pre-cultured in phenol free media in a 8-well chamber (ibidi) pre-coated with biolaminin (BioLamina). The 5x10^3^ cells were seeded per well and DMSO or 500 nM dTAG-V1 treatments were added to the appropriate wells. Prior to imaging, the media was removed and cells were incubated with 100nM of Mitotracker Red FM (Thermo Scientific) in culture medium with DMSO or dTAG-V1 for 30 min. After that, the staining solution was replaced with culture medium and the corresponding treatment. Live imaging was performed on a SP8 Leica confocal microscope. Mitochondria were imaged under a 63x/1.40 oil HC PL APO CS2 objective. Mitotracker Red FM was excited with a white light laser set to 595 nm and emission light was collected with a HyD SMD detector set from 640 to 730 nm. Live-cell imaging of mitochondria in ZFP143-FKBP cells was performed for two biological replicates.

#### Gastruloids

Gastruloids were cultured following a previously established protocol.^146^ In brief, the formation of gastruloids involved the aggregation of mESCs in differentiation medium (N2B27). This medium comprises a combination of DMEM/F12 (Thermo Scientific) and Neurobasal medium (Thermo Scientific), supplemented with 0.5X N2 (Thermo Scientific), 0.5X B27 with retinoic acid (Thermo Scientific), 2mΜ Glutamine, β-mercaptoethanol, and 50U/ml penicillin-streptomycin. U-bottom 96-well plates (Thermo Scientific) were utilised for the culture, and the process was carried out in a humidified incubator set at 5% CO2 and 37ºC. Cell dissociation was performed in N2B27 medium using a serological pipette paired with a 200μL pipette tip. Subsequently, 3.75×104 cells were transferred into N2B27 medium, resulting in a final volume of 5ml. A 40μL aliquot of this suspension, containing approximately 300 cells, was added to each well. At the 48-hour mark, 150μL of N2B27 medium with 3μM CHIR99021 was introduced using the Hamilton Star R&D liquid handling platform. Starting from the following day, the culture medium was replaced with fresh N2B27 medium on a daily basis. For treatment, DMSO or dTAG-V1 at a final concentration of 500nM (in DMSO) were added to the medium.

##### Imaging

Image documentation was carried out using an inverted wide-field microscope (Zeiss Axio Observer Z1 Live) at 72, 96, and 120 hours time points. Images for DMSO and dTAG-V1 treatment at 120 hours were collected for three biological replicates. Images for DMSO and dTAG-V1 time-course treatment were collected for one biological replicate.

##### ATAC-seq

The Tn5 protein was purified by the NKI protein facility as previously described.^74^ To achieve transposon annealing, 10 μL of 10X TE buffer was combined with 45 μL of 100μM Tn5MErev oligonucleotides and 45 μL of 100μM corresponding Tn5ME-A and Tn5ME-B oligonucleotides (listed in Table S1). Subsequently, the adapter solution underwent incubation at 95ºC for 10 minutes and was gradually cooled to 4ºC at a rate of 0.1ºC per second. For transposome formation, the annealed adapters were diluted with an equal volume of H2O. This resulting adapter solution was then mixed with 0.2mg/mL Tn5 at a 1:20 ratio and incubated for 1 hour at 37ºC. The transposomes were either directly utilised for tagmentation or stored at -20ºC for a maximum of 2 weeks.

Gastruloids were collected and dissociated to a single cell suspension by gentle trypsinization. A total of 50,000 cells were gathered in cold PBS and subjected to lysis using a 2x lysis buffer (1 M Tris-HCl pH 7.5, 5 M NaCl, 1 M MgCl2, 10% IGEPAL). Subsequently, the cells were centrifuged, and the resulting pellet was treated with 2xTD buffer (20 mM Tris(hydroxymethyl)aminomethane; 10 mM MgCl2, 20% dimethylformamide, brought at pH 7.6 with acetic acid) and 2 μL of transposon mix. Tagmentation was performed for 1h at 37 C with shaking. After tagmentation, samples were cleaned by adding 9 μl of clean-up buffer (55% buffer 1 (5M NaCl, 0.5M EDTA), 5% SDS and 10mg/ml ProtK). Following this, PCR amplification was conducted twice using KAPA HiFi HotStart PCR ReadyMix (Roche) and P5 and P7 indexed primers (refer to the oligonucleotide list). Fragments ranging from 200 to 700 bp were purified utilising AMPure XP beads (Beckman Coulter), and the quality of the DNA was assessed through Bioanalyzer High Sensitivity DNA analysis (Agilent).

##### Mitochondrial membrane potential assay

Gastruloids were cultured and treated as described above. For collection, gastruloids were trypsinized in N2B27, washed in PBS and further dissociated using a serological pipette paired with a 200 μl pipette tip. Finally, samples were resuspended in N2B27 and divided into three technical replicates. Mitochondrial membrane potential was then measured by using TMRE as described above.

#### Retinoic acid differentiation

For the differentiation using retinoic acid following ZFP143 depletion, ZFP143-FKBP cells were first seeded in normal 2i medium. One day after seeding, DMSO or dTAG-V1 at a final concentration of 500 nM were added to the cells for 24 hours. After that, cells were washed twice with PBS and differentiation medium (N2B27), containing DMEM/F12, supplemented with 0.5X N2 and 0.5X B27, 2mM Glutamine, β-mercaptoethanol and 50U/ml penicillin streptomycin supplemented with *all trans*-retinoic acid (R2625, Sigma-Aldrich) to 1μm final concentration (stock 1:1000 Sigma-Aldrich), was added to the cells. DMSO and dTAG were included to continue the depletion. For harvesting, the cells were dissociated using 2X TVP.

### Quantification and statistical analysis

#### ChIP-seq

##### Data processing

ZFP143 ChIP-seq reads were mapped to the mm10 mouse reference genome assembly using bwa mem v0.7.17-r1188.^120^ Calibrated CTCF ChIP-seq reads were mapped to the mm10 mouse and hg38 human reference genome assemblies using bwa mem v0.7.17-r1188.^120^ Uniquely mapped reads with MAPQ > 10 mapped in proper read pairs (-f 2) were selected using SAMtools v1.12.^121^ Duplicate reads were filtered out using the Picard v2.25.6 “MarkDuplicates” function.

Scaling factors for calibrated CTCF ChIP-seq normalisation were calculated as described previously.^147^ First, to avoid potential bias, CTCF ChIP-seq peaks from the CISTROME database^56^ for E14Tg2A^80^ (ID: 85173, 85177) and HCT116^148^ (ID: 42148, 42149, 42150) were used, and only the peaks found in all biological replicates were extracted. Second, the number of reads covering peaks was calculated using the “multicov” function from BEDTools v2.27.1,^122^ the immunoprecipitation efficiency was calculated as reads covering peaks divided by total number of reads for the mm10 and hg38 genomes, and the fraction of reads mapping to the spike-in hg38 genome was calculated. The absolute scaling factor for each sample was then calculated as the percentage of spike-in chromatin divided by the reads covering peaks for the spike-in genome and normalised by the immunoprecipitation ratio between spike-in and target genomes. Lastly, the relative scaling factors for all samples were calculated by dividing the obtained absolute scaling factors by the highest absolute scaling factor among all samples.

The bigwig coverage tracks for ZFP143 ChIP-seq were generated using the “bamCoverage” function from the deepTools v3.4.2^123^ with the “--effectiveGenomeSize” parameter set to 2652783500 and “--normalizeUsing” parameter set to RPGC. The bigwig coverage tracks for calibrated CTCF ChIP-seq were generated using the “bamCoverage” function from the deepTools v3.4.2^123^ and the calculated relative scaling factors specified under the “--scaleFactor” parameter.

##### Peak calling and analysis

Peak calling was performed using MACS2 v2.2.6.^125^ For ZFP143 ChIP-seq, peaks were called in a narrowPeak mode using input as a control file, with mappable genome size set to mm, q-value cutoff of 0.01, and “--keep-dup” parameter set to all. For CTCF ChIP-seq, peaks were called in a narrowPeak mode using input as a control file, with mappable genome size set to mm, and q-value cutoff of 0.05. Only the peaks detected on the canonical chromosomes outside of the blacklist regions^149^ were retained. Since CTCF ChIP-seq demonstrated little difference between DMSO and dTAG treated conditions, the peaks detected in both cells were pooled together.

Pile-ups of the ChIP-seq signals were calculated using the “computeMatrix” function from deepTools v3.4.2.^123^ Pile-ups were generated in reference-point mode with parameters “--referencePoint” center, “-a” 1000, “-b” 1000, “--missingDataAsZero”, and “--skipZeros”. The resulting matrices were visualised using plotHeatmap function from deepTools v3.4.2. Genomic tracks plots were produced using pyGenomeTracks v3.8.^124^

*De novo* motifs discovery for ZFP143-HA peaks was performed using meme v5.4.1.^126^ The sequences within the peaks were extracted using the “getfasta” function from BEDTools v2.27.1.^122^ MEME was initialised with the parameters “-dna”, “-revcomp”, “-mod” zoops, “-nmotifs” 5 “-minw” 6, “-maxw” 50, “-markov_order” 0. Motif position weight matrices of the *de novo* discovered SBS1 and SBS2 motifs and core collection of JASPAR 2020 motifs in vertebrates^129^^,^^150^ were used to predict motif positions genome-wide with motifmatchr.^127^ For this, “matchMotifs” function was used with background nucleotide frequencies set to “genome”. Motif enrichment in ZFP143-HA peaks was determined using the “permTest” function from regioneR^128^ by evaluating the number of overlaps between peaks and motifs. The background enrichment was assessed using 100 circular permutations of the peaks.

BEDTools v2.27.1 “intersect” function was used to calculate the overlaps between ZFP143-HA peaks and chromHMM states for mESC.^50^ For comparison with publicly available ZFP143 data in mESC, ChIP-seq peaks from the CISTROME database^56^ for J1 mESC^12^ (ID: 36789) were used. The overlap between ZFP143-HA peaks and J1 mESCs peaks was calculated with BEDTools v2.27.1^122^ “intersect” function.

#### Publicly available ChIP-seq data analysis

##### Re-mapping of publicly available ZNF143/ZFP143 datasets

For the systematic re-analysis of the ZNF143/ZFP143 binding, publicly available ZNF143/ZFP143 ChIP-seq datasets from human (N=32) and mouse (N=7) cells were collected (listed in Table S2). Where available, an appropriate input or IgG control was also re-analysed (listed in Table S2). ChIP-seq reads were mapped to the hg38/mm10 mouse reference genome assembly using bwa mem v0.7.17-r1188.^120^ Due to the short read length of the IgG control samples, these data were mapped using bwa aln and bwa samse v0.7.17-r1188.^120^ Uniquely mapped reads with MAPQ > 10 were selected using SAMtools v1.12.^121^ For paired-end datasets, the reads were additionally required to be in proper read pairs (-f 2). Duplicate reads were filtered out using the Picard v2.25.6 “MarkDuplicates” function. Peak calling was performed using MACS2 v2.2.6.^125^ All peaks were called in narrowPeak mode with input or IgG controls where applicable. MACS2 was run with mappable genome size set to hs or mm, q-value cutoff of 0.05, “--keep-dup” set to all, and a “--nomodel” parameter. For datasets where input or IgG control was present, the bigwig coverage tracks were generated using the “bamCompare” function from the deepTools v3.4.2^123^ with the “--operation” parameter set to ratio. For other datasets, the bigwig coverage tracks were generated using the “bamCoverage” function with the appropriate “--effectiveGenomeSize” parameter and “--normalizeUsing” set to RPGC.

##### Co-localization between ZNF143 and CTCF peaks

To analyse the co-localisation between ZNF143/ZFP143 peaks, uniformly processed publicly available CTCF ChIP-seq datasets from the CISTROME database^56^ were used. 512 CTCF peak sets for human and 460 peak sets for mouse were collected. For each ZNF143 dataset, the proportion of ZNF143 peaks overlapping with each CTCF peaks dataset was calculated using the "intersect" function from BEDTools v2.27.1.^122^ Since CTCF binding is relatively conserved, the datasets were not matched according to the cell type. The resulting table showing the overlap between each re-analysed ZNF143/ZFP143 peak set and CTCF, as well as the accession numbers of the CTCF peak set in the CISTROME database, is provided in Mendeley Data.

##### Re-analysis of ZNF143 ChIP-seq in K562 cell line

To explore the overlap between ZNF143 and CTCF peaks, ChIP-seq data generated in the K562 cell line with Proteintech and FLAG antibodies was used for comparison.^57^ As both datasets had biological replicates, a list of reproducible peaks detected by Proteintech and FLAG antibodies within replicates was identified. The reproducible peak sets were then used to classify ZNF143 peaks into common, Proteintech-specific and FLAG-specific categories. Motif enrichment of *de novo* annotated SBS1/SBS2 and CTCF^150^ (JASPAR ID: MA0139.1) motifs in common, Proteintech-specific and FLAG-specific peaks was calculated with “permTest” function from regioneR^128^ as described above. To compare signals detected by different antibodies, the ZNF143 dataset produced with a custom antibody^12^ and the CTCF dataset from ENCODE^57^ with corresponding controls (see Table S2) were re-processed as described above. The pile-ups of the ChIP-seq signals were calculated using the “computeMatrix” function from deepTools v3.4.2^123^ as described above.

To reproduce the enrichment analysis at the loop anchors identified in the K562 cell line, the loop annotation from the original study was used.^40^ In addition, peak datasets for 11 histone modifications, 144 transcription factors, and 1 DHS dataset from ENCODE used in the original publication^40^ were downloaded (listed in Table S3 of the original study). As loop annotation and peak sets were for the hg19 human reference genome assembly, the coordinates of the common, Proteintech-specific and FLAG-specific ZNF143 peaks were lifted over from the hg38 to the hg19 assembly using liftOver. Enrichment was then calculated using the “permTest” function from regioneR^128^ by evaluating the number of overlaps between loop anchors and peaks and using 100 circular permutations to estimate the background. To get the enrichment scores relative to other factors, the enrichments were normalised by dividing all enrichments by the average enrichment, as described in the original study. Proteintech-specific, FLAG-specific, and common peaks were excluded from the mean calculation and the calculated value was retrospectively applied to these datasets to obtain a relative enrichment score. The fraction of loop anchors containing the peaks was calculated using the “intersect” function from BEDTools v2.27.1.^122^

##### CTCF binding to chromatin is independent of ZNF143

A study by Zhou et al. previously suggested that a conditional knockout of ZFP143 in haematopoietic stem and progenitor cells affects CTCF binding.^38^ The authors reported that in the ZFP143 knockout (KO) condition, CTCF binding was reduced at the majority of CTCF peaks (referred to as ZFP143-related peaks, N=27,801), although some retained the same signal as in the wild-type (WT) condition (referred to as ZFP143-unrelated peaks, N=4196). To investigate the cause of this observation, CTCF ChIP-seq data in the WT and KO cells was re-analysed.

In this study, two independent biological replicates of CTCF ChIP-seq were performed and deposited. Therefore, ChIP-seq CTCF signals from both replicates were used to generate heatmaps over ZFP143-related and ZFP143-unrelated regions. Surprisingly, the loss of signal in the CTCF ChIP-seq dataset over the ZFP143-related peaks could only be detected in the first of the two replicates (Figure S4A). In the second biological replicate, the signal in WT was very similar to the signal in both KO replicates, suggesting potential differences between two CTCF ChIP-seq datasets in the WT, but not KO condition. To understand the nature of this replicate-specific behaviour, orthogonal CTCF ChIP-seq datasets from the same cell type^151^^,^^152^ were added. Heatmaps over the same regions revealed that both datasets have very weak CTCF signals over ZFP143-related regions, similar to the CTCF signal from the second WT replicate (Figure S4B). In addition, CTCF motif analysis revealed that CTCF motifs are weak or absent in the ZFP143-related peaks, in contrast to the ZFP143-unrelated peaks, where CTCF motifs have about average strength (Figure S4C). Furthermore, overlapping these two sets of peaks with the compendium of CTCF peaks from CISTROME revealed that only 30% of the ZFP143-related peaks show overlap, whereas this fraction is almost 100% for the ZFP143-unrelated peaks (Figure S4D).

It has been previously reported that ChIP-seq may be subject to GC bias, in some cases leading to a higher proportion of false positive peaks.^153^ To investigate whether this might be the cause of the differences between CTCF replicates in WT, the observed versus expected read counts for different GC content bins were calculated. This analysis revealed a severe skew towards a higher than expected number of GC-rich reads in the first replicate of CTCF ChIP-seq in the WT condition, but not in the second replicate and neither of the KO samples, suggesting that an extreme GC bias is present (Figures S4E and S4F).

Taken together, this analysis suggests that the differences in signal in the CTCF ChIP-seq upon ZFP143 knockout are likely a result of GC bias in one of the CTCF ChIP-seq replicates, rather than a true biological change induced by the loss of ZFP143. As only this replicate was used to draw conclusions in the original study, the identified set of ZFP143-related peaks is likely an artefact of the peak calling procedure. The low overlap with a compendium of CTCF peaks and the absence of strong CTCF motifs further suggest that these regions are unlikely to represent actual CTCF binding sites.

##### ZNF143 binding to chromatin is independent of CTCF

Another study by Zhang et al. has recently suggested that acute depletion of CTCF in the endometrial carcinoma cell line HEC1B leads to changes in ZNF143 occupancy.^39^ The signal in the ZNF143 ChIP-seq dataset was found to be reduced at CTCF binding sites, whereas it was maintained at loci where only ZNF143 signal was present, but not CTCF signal. To investigate this further, we re-analysed the ChIP-nexus signals for ZNF143 and CTCF using the peak coordinates deposited by the authors.

Peaks in the ZNF143 ChIP-nexus dataset from the control HEC1B cells were classified as ZNF143-only (N=15377) or shared (N=19745) based on overlap with CTCF peaks from the same experimental condition using the intersect function of BEDTools v2.27.1.^122^ ZNF143 ChIP-nexus signals in control and CTCF-depleted conditions were then used to generate heat maps over the ZNF143-only and shared peaks (Figures S4G and S4H). As reported in the original study, a decrease in ZNF143 signal was observed only for the shared sites. However, the antibody used for ChIP-nexus is the polyclonal anti-ZNF143 Proteintech 16618-1-AP antibody, which we believe to be cross-reacting with CTCF. Therefore, the most parsimonious explanation why ZNF143 signal is lost at CTCF sites is the recognition of CTCF by the Proteintech antibody in the untreated condition and its absence upon CTCF depletion.

This finding suggests that it is unlikely that CTCF positions ZNF143 at CTCF binding sites. The data from Zhou et al. suggest that ZNF143 positions CTCF on chromatin, whereas the data from Zhang et al. suggest that CTCF positions ZNF143 on chromatin, albeit at different positions in the genome. Our interpretation of these data is that neither protein affects the positioning of the other, which is in line with our own observation in the acute ZFP143 depletion setting. We would like to emphasise that the independence of CTCF and ZFP143 in chromatin binding and gene regulation is also found in the degron lines from our study and accompanying study.^79^

##### ZNF143-CTCF motif pairs positioning is an artefact of repetitive elements

Another observation that could indicate a cooperative role for ZFP143 and CTCF chromatin binding is that approximately 60% of ZFP143 motifs were found to be located exactly 37 bp away from the nearest CTCF motif in the mouse genome.^38^ ZFP143-CTCF motif pairs provided by the authors were utilised to plot the CTCF and ZFP143 occupancy from the study that reported the motif pairs at these positions. To our surprise, no signal could be detected for either CTCF or ZFP143 in any of the replicates (Figure S4I). To understand whether a specific feature is associated with these motif pairs, a number of genomic characteristics were examined. Strikingly, the reported CTCF-ZFP143 motif pairs completely overlapped with SINE/B2 repetitive elements (Figure S4J). This suggests that the proximity of CTCF and ZFP143 motifs is a chance co-occurrence found in this specific class of repeat elements. The fact that these motif pairs are not bound by either protein makes it unlikely that CTCF or ZFP143 exert any regulatory function at these repeat elements, either in gene regulation or 3D genome organisation.

#### HEK293T ChIP-seq data analysis

CTCF ChIP-seq and ZNF143 ChIP-seq generated with Proteintech (this study) and HA^58^ antibodies were mapped to the hg38 mouse reference genome assembly using bwa mem v0.7.17-r1188.^120^ Uniquely mapped reads with MAPQ > 10 mapped in proper read pairs (-f 2) were selected using SAMtools v1.12.^121^ Duplicate reads were filtered out using the Picard v2.25.6 “MarkDuplicates” function. The bigwig coverage tracks for were generated using the “bamCoverage” function from the deepTools v3.4.2^123^ with the “--effectiveGenomeSize” parameter set to 2913022398 and “--normalizeUsing” parameter set to RPGC.

Peak calling was performed using MACS2 v2.2.6^125^ for ZNF143 ChIP-seq data. Peaks were called in a narrowPeak mode, with mappable genome size set to hs, q-value cutoff of 0.01, “--keep-dup” set to all, and a “--nomodel” parameter. Only the peaks detected on the canonical chromosomes outside of the blacklist regions^149^ were retained. Reproducible peaks detected by Proteintech antibody were identified by taking peaks detected in each of the 4 replicates and any of the 4 replicates in untreated and dTAG-V1 treated conditions, respectively. Reproducible peaks detected by HA antibody were identified by taking peaks detected in each of the 2 replicates in both untreated and dTAG-V1 treated conditions. These lists were then merged to obtain two lists of peaks detected by Proteintech and HA antibodies.

Peak sets detected by Proteintech and HA antibodies were then used to classify ZNF143 peaks into common, Proteintech-specific and HA-specific categories. Motif enrichment of *de novo* annotated SBS1/SBS2 and CTCF^150^ (JASPAR ID: MA0139.1) motifs in common, Proteintech-specific and HA-specific peaks was calculated with “permTest” function from regioneR^128^ as described above. To compare signals detected by different antibodies, the ZNF143 dataset produced with a custom antibody^12^ (see Table S2) was re-processed as described above. The pile-ups of the ChIP-seq signals were calculated using the “computeMatrix” function from deepTools v3.4.2^123^ as described above.

#### ZFP143 targets analysis

The following procedure was developed to annotate ZFP143-bound genes. All transcripts corresponding to the expressed protein-coding and long non-coding RNA ("transcript_type" field is protein-coding, lincRNA, processed_transcript, bidirectional_promoter_lncRNA or antisense) with high confidence levels ("transcript_support_level" field is 1, 2, 3, 4 or 5) from the GENCODE vM25 gene annotation^154^ were extracted. For each transcript, the coordinates of the TSS window were calculated by taking the coordinates 2 kb upstream and 1 kb downstream of the annotated TSS. The transcripts for which the TSS window overlapped the ZFP143 peaks and the distance between the TSS and the peak centre was less than 2 kb were retained. Next, for each gene, the transcript that met most of the following ranked criteria was retained: highest transcript support level, shortest distance to peak, highest expression, and maximum length. Lastly, when the same ZFP143 peak was associated with multiple genes on the same strand, the more appropriate gene was assigned based on (i) transcript type, (ii) transcript support level, and (iii) presence of genes with lower annotation quality at the same location. In particular, protein-coding genes were preferred over lincRNAs, and lincRNAs were preferred over all other types of non-coding RNAs. Genes with transcript support levels of 1 or 2 were prioritised over the others. Finally, genes without canonical names were given a lower priority than any other. If the application of these three rules did not resolve the ambiguous assignment of the ZFP143-bound gene, both genes were discarded.

The described procedure resulted in N=2226 genes being annotated as ZFP143-bound, of which N=1975 were protein-coding genes and N=251 were non-coding RNA. On the other hand, N=1846 ZFP143 peaks were assigned to genes, of which N=375 were located at bidirectionally transcribed promoters. Gene ontology analysis of ZFP143-bound genes was performed using the GO term over-representation test from g:Profiler.^130^ The annotation of genes with GO terms from Ensembl (release 110) was utilised, and electronic GO annotations were removed prior to the analysis. An FDR threshold of 0.05 was applied to filter significantly enriched GO terms.

##### Conserved human and mouse ZNF143/ZFP143-bound genes

To analyse the conservation of ZNF143/ZFP143 binding, re-analysed ChIP-seq peaks from human and mouse cell lines were used. All identified peaks for the human and mouse datasets were pooled together and for each peak the number of samples in which the peak was detected was calculated using the “count_overlaps” function from bioframe v0.3.3.^132^ The datasets were then classified according to the cell type (32 datasets mapped to 16 cell types in human, 7 datasets mapped to 5 cell types in mouse, Table S2). ZNF143 binding sites were considered conserved across human datasets if a peak was identified in every cell type. The same procedure was followed for mouse ZFP143 datasets. This resulted in N=1456 conserved peaks in human and N=1240 conserved peaks in mouse. 99.5% and 97.5% of the conserved human and mouse peaks, respectively, contained SBS1 and SBS2 motifs, indicating that these are strong ZNF143/ZFP143 binding sites. To assign conserved ZNF143/ZFP143-bound genes, the procedure described above for ZFP143-HA peaks was used, and N=1696 and N=1504 conserved ZNF143/ZFP143-bound genes were annotated in human and mouse, respectively. To map the mouse orthologs of conserved ZNF143-bound human genes, biomaRt^131^ with Ensembl genes (release 110) was used. Gene ontology analysis of the conserved ZNF143/ZFP143-bound genes was performed using the GO term over-representation test from g:Profiler.^130^ The annotation of genes with GO terms from Ensembl (release 110) was utilised, and electronic GO annotations were removed prior to the analysis. An FDR threshold of 0.05 was applied to filter significantly enriched GO terms.

#### Hi-C

Hi-C reads were mapped to the mm10 mouse reference genome assembly using bwa mem v0.7.17-r1188^120^ with the Open2C distiller-nf pipeline (https://github.com/open2c/distiller-nf). Mapped reads were parsed using pairtools v0.3.0^133^ with the “walks-policy” parameter set to “mask” and the “max_mismatch_bp” parameter set to 1. Read pairs were then binned into Hi-C contact matrices and written as.cool files. Iterative correction of the matrices and removal of low coverage bins was performed using the "balance" function from cooler v0.8.11.^134^

Genome-wide relative contact probability curves and their derivatives were calculated for the 5 kb resolution Hi-C contact matrices using cooltools v0.5.1.^135^ Expected contact matrices were obtained using the "compute-expected" function from cooltools with the "ignore-diags" parameter set to 0. Average loops were calculated for 5 kb resolution observed-over-expected Hi-C contact matrices using coolpup.py v0.9.5^136^ with "pad" set to 200 and "min-dist" set to 0. Loops previously annotated in high-resolution Hi-C and Micro-C datasets^52^^,^^53^ were used for the average loop calculations.

#### 4C-seq

4C-seq reads were processed as previously described.^55^^,^^84^ First, the restriction enzyme (RE) fragment map of the mm10 mouse reference genome was generated for DpnII and Csp6I REs. The fragments derived from mitochondrial DNA and the so-called blind fragments, whose ends were cut by the same restriction enzyme, were removed. Secondly, the sequencing data were demultiplexed per viewpoint by scanning the forward and reverse reads for viewpoint-specific primers, allowing for one mismatch. The forward reads were then trimmed to the start of the DpnII cut site. The resulting reads per viewpoint were mapped to the mm10 mouse reference genome assembly using the bwa aln and bwa samse v0.7.17-r1188.^120^ To discard multi-mapping reads, only those with MAPQ > 3 were retained using SAMtools v1.12.^121^ Finally, to generate the read counts per RE fragment, for each fragment the number of mapped reads that have the same start or end coordinates as the fragment was counted. To obtain 4C contact profiles, the counts were normalised to the total number of overlaps detected per million reads.

#### TT-seq

TT-seq reads were mapped to the mm10 mouse reference genome assembly using STAR v2.7.9a^137^ with GENCODE vM25 gene annotation.^154^ Uniquely mapped reads with MAPQ > 10 were selected using SAMtools v1.12.^121^ Duplicate reads were filtered out using the Picard v2.25.6 “MarkDuplicates” function. The reads were split by strand using SAMtools as described previously.^51^ Forward strand reads were extracted by including (i) reads that are second in read pair (-f 128), excluding reads that are mapped to the reverse strand (-F 16) and (ii) reads that are first in a pair and are mapped to the reverse strand (-f 80). Reverse strand reads were extracted by including (i) reads that are first in read pair (-f 64), excluding reads that are mapped to the reverse strand (-F 16) and (ii) reads that are second in a pair and are mapped to the reverse strand (-f 144). Gene counts were obtained using the ”htseq-count” function from HTSeq v0.13.5.^138^ Counts were calculated separately for genes from forward and reverse strands with the parameters “--stranded no”, “--nonunique all”, “--order pos”, and “--type gene”. Bigwig coverage tracks were generated separately for forward and reverse strands using the deepTools v3.4.2^123^ with the “bamCoverage” function. Scale factors for bigwig generation were calculated as the number of reads per sample adjusted to match the coverage of the sample with the highest coverage. Genomic tracks plots were produced using pyGenomeTracks v3.8.^124^

Differentially expressed genes were identified using the DESeq2 v1.30.1.^139^ Low expressed genes were filtered by requiring half of the samples to have gene counts greater than 10. The “nbinomWaldTest” function with default parameters was used to test contrasts of dTAG-V1 treatment time against DMSO. Genes with FDR < 0.05 and absolute log_2_-fold-change > 0.5 were considered differentially expressed. MitoCarta v3.0^60^ was used to annotate the differentially expressed genes with the mitochondrial localisation, pathways, and processes. Gene set enrichment analysis was performed using GSEA v4.3.2^140^ in pre-ranked mode with 10,000 permutations and gene sets from MSigDB v2023.2.^155^ Gene sets with FDR < 0.05 were considered significant.

#### Mass spectrometry

Raw data were analysed by DIA-NN v1.8^141^ without a spectral library and with the “Deep learning” option enabled. The Swissprot Mus Musculus database (17.125 entries, release 2022_08) was added for the library-free search. The quantification strategy was set to Robust LC (high accuracy) and the MBR option was enabled. The other settings were kept at the default values. The protein groups report from DIA-NN was used for downstream analysis in Perseus v2.0.10.0.^142^ Values were log_2_-transformed, after which proteins were filtered for at least 100% valid values in at least one sample group. Missing values were replaced by imputation based on a normal distribution with a width of 0.3 and a minimal downshift of 2.4. Differentially expressed proteins were determined using a Student’s t test (minimal threshold: -log(*p* value) ≥ 1.3 and [x-y] ≥ 0.5 | [x-y] ≤ -0.5).

Gene set enrichment analysis was performed using GSEA v4.3.2^140^ in pre-ranked mode with 10,000 permutations and gene sets from MSigDB v2023.2.^155^ Genes corresponding to the identified proteins were pre-ranked by the protein abundance difference derived from differential expression analysis. Gene sets with FDR < 0.05 were considered significant.

#### RNA-seq

RNA-seq reads were mapped to the mm10 mouse reference genome assembly using STAR v2.7.9a^137^ with GENCODE vM25 gene annotation.^154^ Read counts per gene were obtained using the "quantMode" parameter in STAR. Differentially expressed genes were identified using the DESeq2 v1.30.1.^139^ Low expressed genes were filtered by requiring half of the samples to have gene counts greater than 10. Distances between the samples were calculated based on the VST-transformed gene count matrix using the “plotPCA” function. The “nbinomWaldTest” function with default parameters was used to test contrasts. Test results at the lfcThreshold of log_2_(1.5) were extracted and used for downstream analyses. Genes with FDR < 0.01 and absolute log_2_-fold-change > 0 were considered differentially expressed. Gene set enrichment analysis was performed using GSEA v4.3.2^140^ in pre-ranked mode with 10,000 permutations and gene sets from MSigDB v2023.2.^155^ Gene sets with FDR < 0.1 were considered significant.

#### ATAC-seq

ATAC-seq reads were mapped to the mm10 mouse reference genome assembly bwa mem v0.7.17-r1188.^120^ Uniquely mapped reads that were not mapped to mitochondrial DNA and had MAPQ > 10 were selected using SAMtools v1.12.^121^ Duplicate reads were filtered out using the Picard v2.25.6 “MarkDuplicates” function.

To deconvolve cell type compositions in bulk ATAC-seq samples from control and ZFP143-depleted gastruloid, previously published single-cell ATAC-seq atlas of gastruloid development was used.^74^ Firstly, pseudobulk ATAC-seq profiles for each cell type (cluster) were generated from single-cell data by summing counts in each peak across all cells belonging to the same clusters. Then, the read counts of DMSO and dTAG-V1 treated bulk samples in the same peak set used in the single-cell data were generated. Bulk and pseudobulk counts were concatenated, normalised using the median of ratios normalisation implemented in DESeq2 v1.30.1,^139^ and transformed using the variance stabilising transform (VST).

Since bulk samples can be thought of as linear combinations of different cell types, independent component analysis (ICA) can be used to unmix the contributions of constituent cell types in bulk samples. ICA is a method for separating a multivariate signal into a predefined number of additive, mutually independent components. Briefly, ICA was performed on VST-transformed and zero-centred pseudobulk counts, using the FastICA algorithm, implemented in the “icafast” function from ica R package, with number of components set to 7 and a maximum of 100 algorithm iterations. The source signal estimates and the unmixing matrix obtained from ICA were subsequently used on the VST-transformed bulk count matrix to unmix the cell type contributions. Finally, the cell type proportion estimates were obtained by setting the negative contributions to zero and normalising contributions to the sum of the contributions for each sample.

#### Image analysis

Gastruloid brightfield images were analysed using ImageJ v2.9.0^156^ to quantify morphological features. Files were opened using Bio-formats.^157^ Images were pre-processed by applying a Gaussian filter (sigma=3) to smooth the images and then the local contrast was normalised (x=200, y=200, STD=3, centered). The gastruloid outline was selected using a supervised thresholding, converted into a binary mask, and consequently analysed with the “Analyse particles” plugin. Briefly, the gastruloid outline is selected as a region of interest (ROI) and shape descriptors, such as “Roundness”, “Aspect ratio”, and “Area” were calculated.

## Supplemental information

Document S1. Figures S1–S11 and supplemental references

Table S1. List of the oligonucleotides and primers used in this study, related to STAR Methods

Table S2. List of the publicly available datasets reanalyzed in this study, related to STAR Methods

Document S2. Article plus supplemental information
