# Supplementary material for: ZNF143 is a transcriptional regulator of nuclear-encoded mitochondrial genes that acts independently of looping and CTCF
Source: Mol Cell. 2025 Jan 2;85(1):24–41.e11. doi: 10.1016/j.molcel.2024.11.031 (PMC11687419; doi:10.1016/j.molcel.2024.11.031)
Supplement: Document S1. Figures S1–S11 and supplemental references [file mmc1.pdf]

Molecular Cell, Volume *85*

Supplemental information

ZNF143 is a transcriptional regulator of nuclear-encoded mitochondrial genes that acts independently of looping and CTCF

Mikhail D. Magnitov, Michela Maresca, Noemí Alonso Saiz, Hans Teunissen, Jinhong Dong, Kizhakke M. Sathyan, Luca Braccioli, Michael J. Guertin, and Elzo de Wit

Figure S1

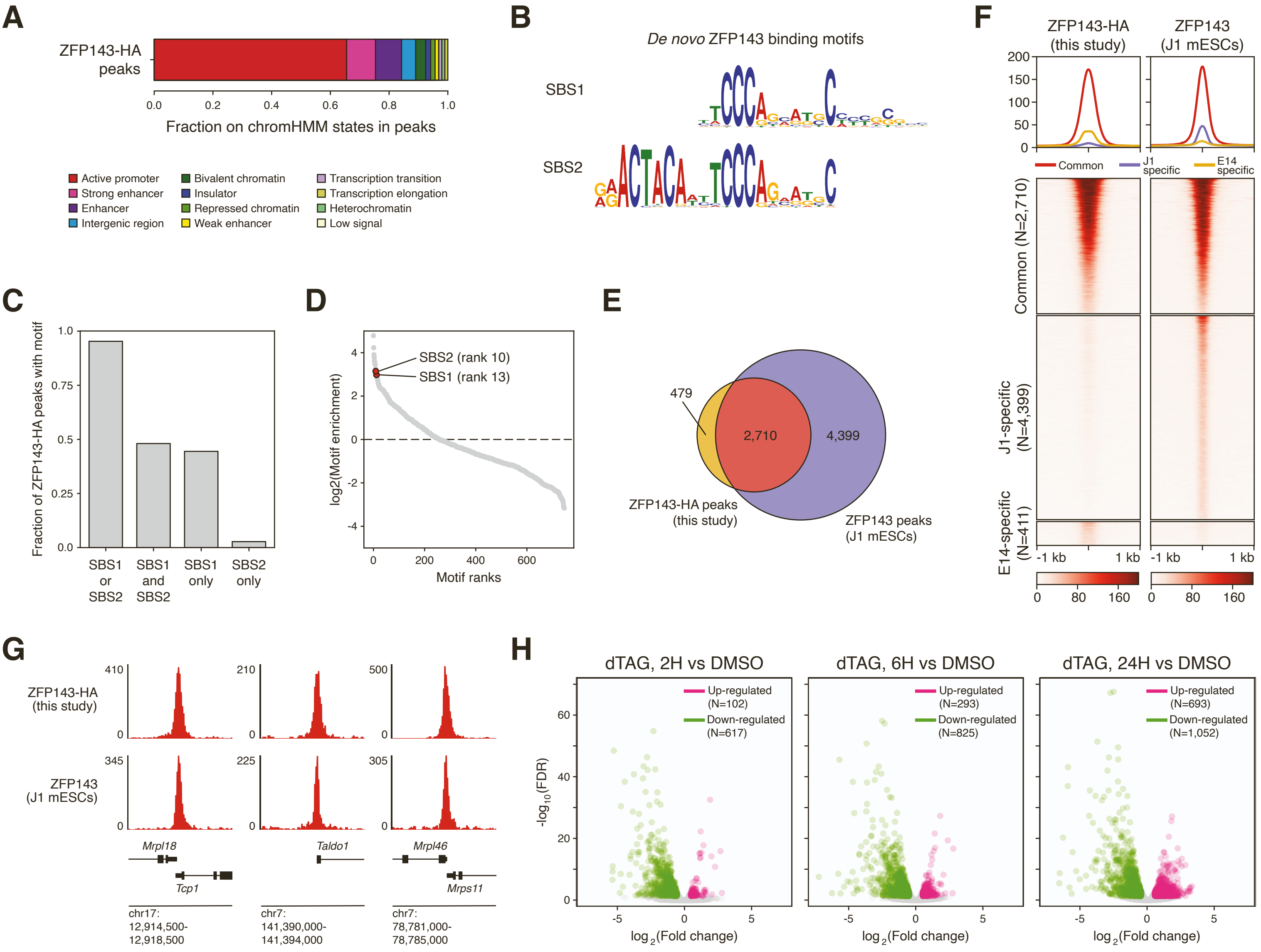
A
ZFP143-HA peaks
Fraction on chromHMM states in peaks
Active promoter
Strong enhancer
Enhancer
Intergenic region
Bivalent chromatin
Insulator
Repressed chromatin
Weak enhancer
Transcription transition
Transcription elongation
Heterochromatin
Low signal
B
De novo ZFP143 binding motifs
SBS1
SBS2
C
Fraction of ZFP143-HA peaks with motif
SBS1 or SBS2
SBS1 and SBS2
SBS1 only
SBS2 only
D
log2(Motif enrichment)
SBS2 (rank 10)
SBS1 (rank 13)
Motif ranks
E
479
2,710
4,399
ZFP143-HA peaks (this study)
ZFP143 peaks (J1 mESCs)
F
ZFP143-HA (this study)
ZFP143 (J1 mESCs)
Common
J1 specific
E14 specific
Common (N=2,710)
J1-specific (N=4,399)
E14-specific (N=411)
-1 kb
1 kb
G
ZFP143-HA (this study)
ZFP143 (J1 mESCs)
Mrpl18
Tcp1
Taldo1
Mrpl46
Mrps11
chr17: 12,914,500- 12,918,500
chr7: 141,390,000- 141,394,000
chr7: 78,781,000- 78,785,000
H
dTAG, 2H vs DMSO
dTAG, 6H vs DMSO
dTAG, 24H vs DMSO
Up-regulated (N=102)
Down-regulated (N=617)
Up-regulated (N=293)
Down-regulated (N=825)
Up-regulated (N=693)
Down-regulated (N=1,052)
-log10(FDR)
log2(Fold change)

Figure S1. ChIP-seq and TT-seq in the ZFP143-FKBP cell line recapitulate known patterns of ZFP143 binding and gene regulation, related to Figure 1. (A) Distribution of ZFP143-HA ChIP-seq peaks across chromatin states annotated in mESCs[S1]. Colours represent different chromatin states as in the original annotation. **(B)** *De novo* annotated SBS1 and SBS2 motifs in ZFP143-HA ChIP-seq peaks. **(C)** Fraction of ZFP143-HA peaks containing annotated SBS1 and SBS2 motifs. **(D)** Global motif enrichment analysis in ZFP143-HA peaks using the *de novo* annotated SBS motifs and motifs from the JASPAR database[S2]. **(E)** Venn diagram showing the overlap between ZFP143-HA peaks and endogenous ZFP143 peaks detected in J1 mESCs with a custom antibody recognizing the endogenous ZFP143[S3]. **(F)** Tornado plots of ChIP-seq signals for ZFP143-HA in E14 mESCs and endogenous ZFP143 in J1 mESCs[S3] centred at common, J1-specific and E14-specific peaks. **(G)** Genomic tracks showing ChIP-seq signals of ZFP143-HA in E14 mESCs and endogenous ZFP143 in J1 mESCs[S3] at ZFP143 target loci, *Tcp1*, *Taldo1*, and *Mrps11*. **(H)** Volcano plots showing effect sizes and significance of the down-regulated (green) and up-regulated (pink) genes measured by TT-seq in mESCs after dTAG-V1 treatment, compared to DMSO treatment. The number of differentially expressed genes is indicated in the top right corners.

Figure S2

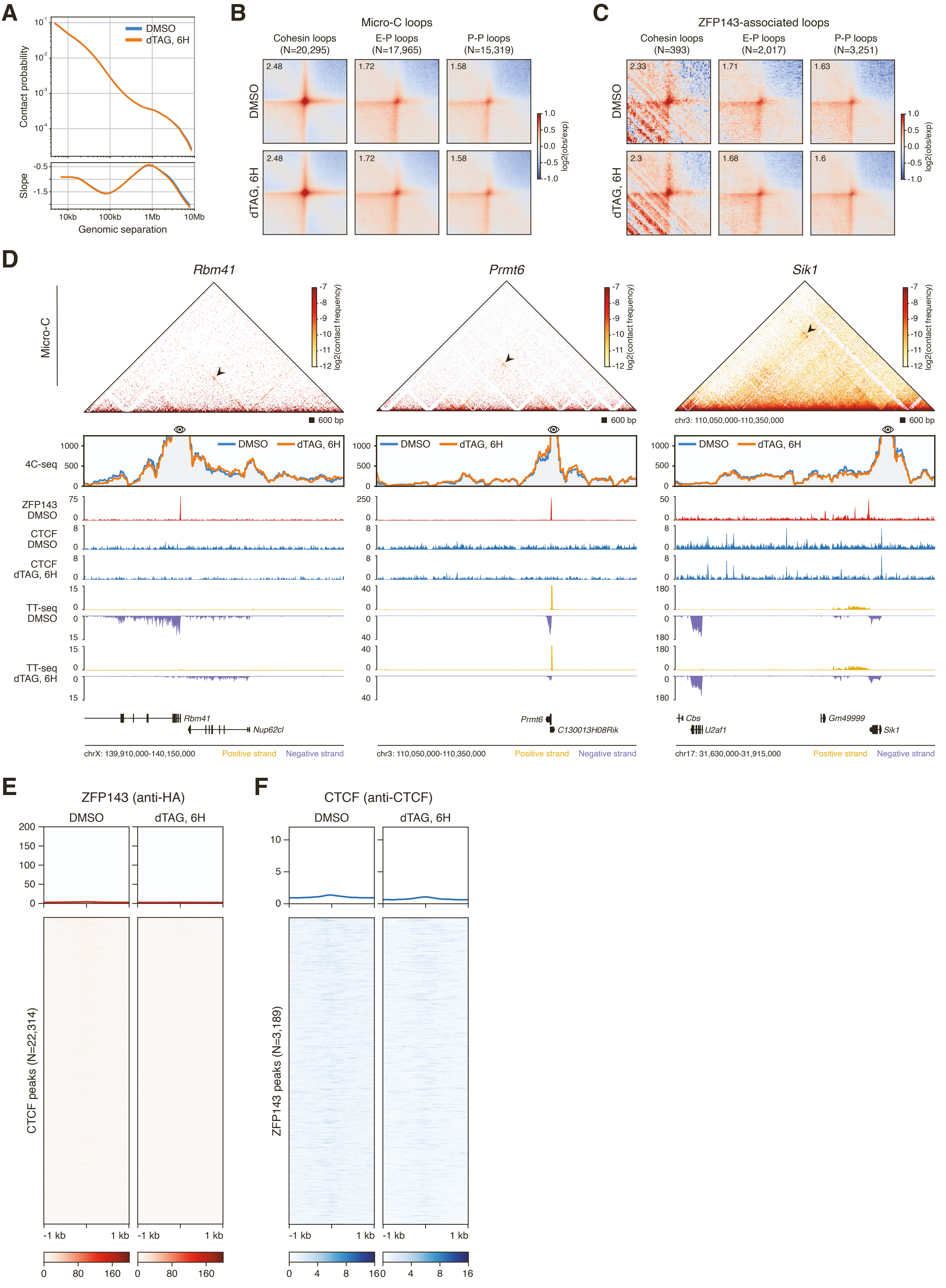

A
Contact probability
DMSO
dTAG, 6H
Slope
10kb
100kb
1Mb
10Mb
Genomic separation
B
Micro-C loops
Cohesin loops (N=20,295)
E-P loops (N=17,965)
P-P loops (N=15,319)
DMSO
dTAG, 6H
2.48
1.72
1.58
2.48
1.72
1.58
log2(obs/exp)
C
ZFP143-associated loops
Cohesin loops (N=393)
E-P loops (N=2,017)
P-P loops (N=3,251)
2.33
1.71
1.63
2.3
1.68
1.6
D
Rbm41
Prmt6
Sik1
Micro-C
log2(contact frequency)
600 bp
chr3: 110,050,000-110,350,000
4C-seq
ZFP143 DMSO
CTCF DMSO
CTCF dTAG, 6H
TT-seq DMSO
TT-seq dTAG, 6H
Rbm41
Nup62cl
Prmt6
C130013H08Rik
Cbs
U2af1
Gm49999
Sik1
chrX: 139,910,000-140,150,000
chr3: 110,050,000-110,350,000
chr17: 31,630,000-31,915,000
Positive strand
Negative strand
E
ZFP143 (anti-HA)
DMSO
dTAG, 6H
CTCF peaks (N=22,314)
-1 kb
1 kb
F
CTCF (anti-CTCF)
ZFP143 peaks (N=3,189)

Figure S2. Chromatin interactions are largely unperturbed following ZFP143 depletion, related to Figure 2. (A) Relative contact probability plot (top panel) and its derivative (bottom panel) calculated from the Hi-C matrices of DMSO (blue) and dTAG-V1 (orange) treated cells. **(B)** Average cohesin (left), enhancer-promoter (E-P, middle) and promoter-promoter (P-P, right) loops[S4] in DMSO and dTAG-V1 treated cells. Value in the upper-right corner indicates the interaction strength of the loop over the background. **(C)** Same as in (B) but for the average ZFP143-associated loops (containing ZFP143 peak in at least one loop anchor). **(D)** High-resolution 4C-seq data generated for the ZFP143-bound genes *Rbm41* (left panel) and *Prmt6* (middle panel), and non-ZFP143-bound control gene *Sik1* (right panel), using gene promoters as viewpoints. The matrix in the top panel represents interaction frequencies in a previously published high-resolution Micro-C dataset[S5]. The arrows point to detected Micro-C chromatin loops. The bottom panel shows 4C contact profiles in DMSO (blue) and in dTAG-V1 (orange) treated cells. Genomic tracks show ZFP143-HA ChIP-seq (red), calibrated CTCF ChIP-seq (blue), TT-seq nascent transcription (yellow for sense and purple for antisense transcription) in control and dTAG-V1 treated cells. **(E)** Tornado plots of ZFP143-HA ChIP-seq signal centred at CTCF peaks in DMSO and dTAG-V1 treated cells. **(F)** Same as in (E) but for the calibrated CTCF ChIP-seq signal centred at ZFP143-HA peaks.

Figure S3

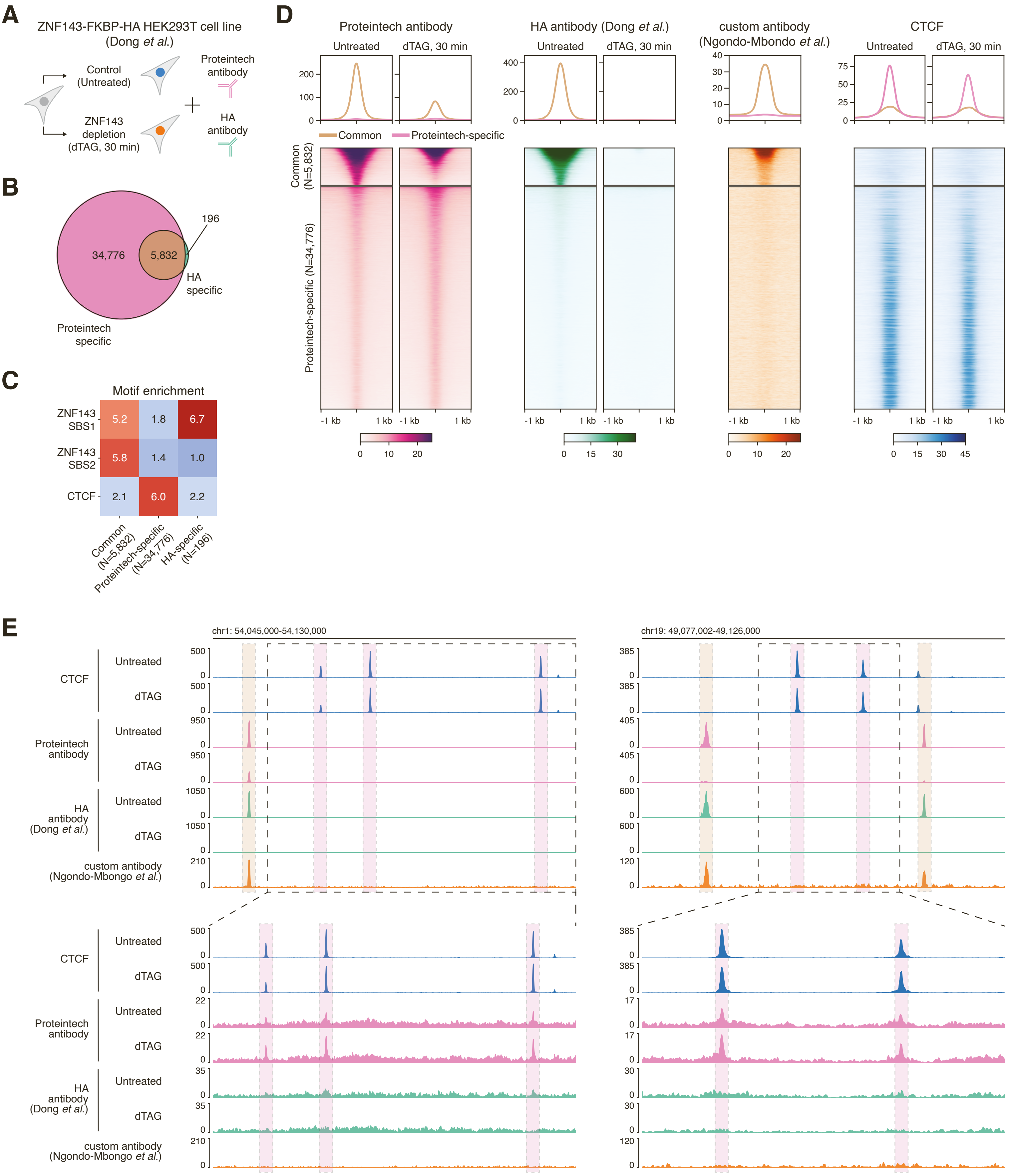
A
ZNF143-FKBP-HA HEK293T cell line (Dong et al.)
Control (Untreated)
ZNF143 depletion (dTAG, 30 min)
Proteintech antibody
HA antibody
B
34,776
5,832
196
HA specific
Proteintech specific
C
Motif enrichment
ZNF143 SBS1
ZNF143 SBS2
CTCF
5.2
1.8
6.7
5.8
1.4
1.0
2.1
6.0
2.2
Common (N=5,832)
Proteintech-specific (N=34,776)
HA-specific (N=196)
D
Proteintech antibody
HA antibody (Dong et al.)
custom antibody (Ngondo-Mbondo et al.)
CTCF
Untreated
dTAG, 30 min
Common
Proteintech-specific
Common (N=5,832)
Proteintech-specific (N=34,776)
-1 kb
1 kb
E
chr1: 54,045,000-54,130,000
chr19: 49,077,002-49,126,000
CTCF
Proteintech antibody
HA antibody (Dong et al.)
custom antibody (Ngondo-Mbongo et al.)
Untreated
dTAG

Figure S3. ChIP-seq data in HEK293T cells confirms ZNF143 antibody cross-reactivity with CTCF, related to Figure 3. (A) Schematic of the ChIP-seq experiment in HEK293T cell line. **(B)** Venn diagram showing the overlap between ZNF143 peaks detected by Proteintech (light pink) and HA[S6] (light green) antibodies in HEK293T cells. **(C)** Heatmap showing the enrichment of SBS (i.e. ZNF143) and CTCF motifs in common, Proteintech-specific, and HA-specific peaks in HEK293T cells. **(D)** Tornado plots of ChIP-seq signals detected by Proteintech (light pink), HA[S6] (light green), and custom[S3] (orange) antibodies, and CTCF signal (blue) in HEK293T cells. The ChIP-seq signals are centred on common (top) and Proteintech-specific (bottom) peaks. **(E)** Genomic tracks showing ChIP-seq signals for CTCF (blue) and signals detected by Proteintech (pink), HA[S6] (light green), and custom[S3] (orange) antibodies in HEK293T cells. Rectangles indicate common (highlighted in brown) and Proteintech-specific (highlighted in pink) peaks in the regions. The bottom tracks represent zoomed in regions with the Proteintech-specific peaks. Note the increase of ChIP-seq signal detected by the Proteintech antibody at the CTCF sites in the dTAG-V1 treated condition.

Figure S4

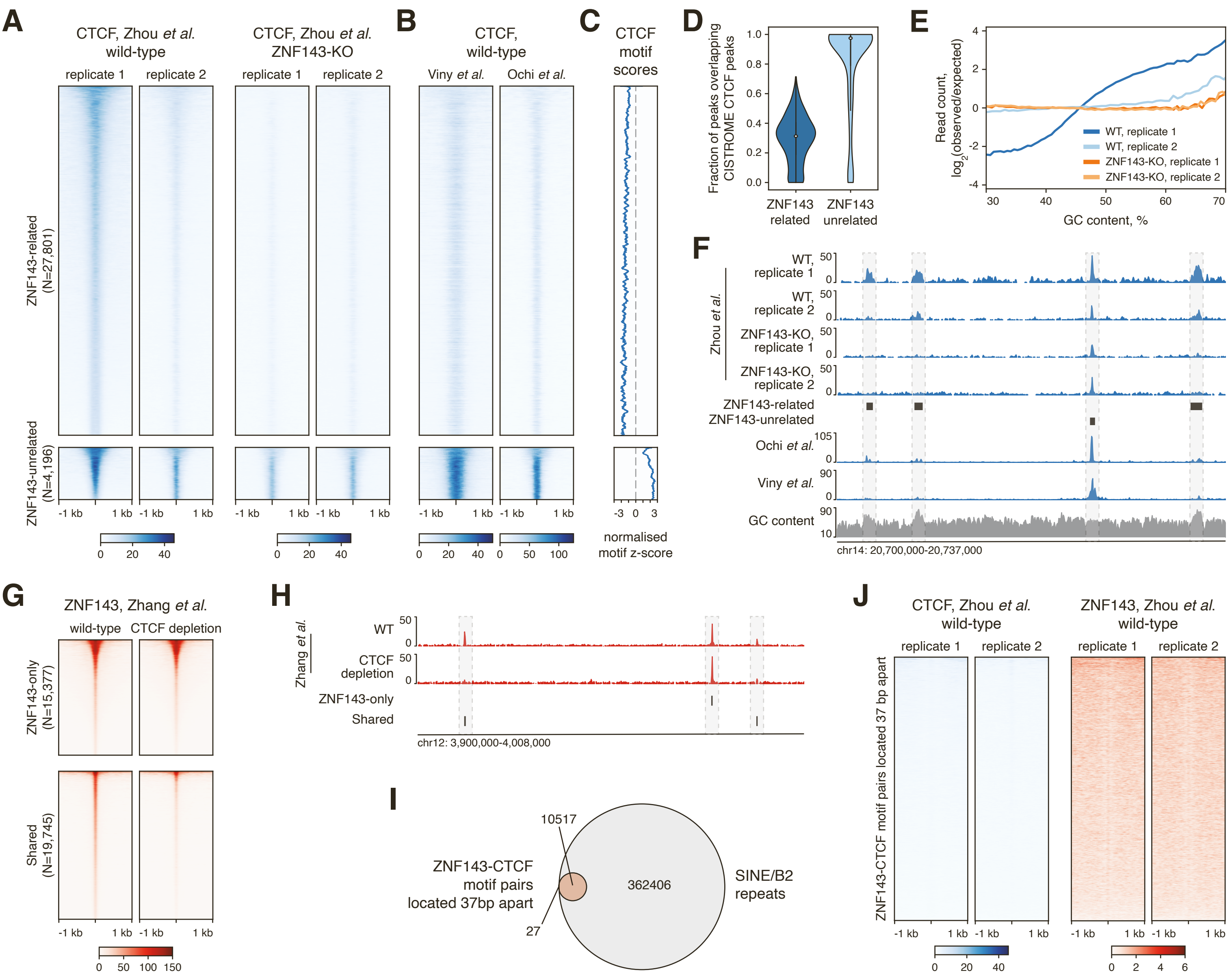

A
CTCF, Zhou et al. wild-type
replicate 1
replicate 2
CTCF, Zhou et al. ZNF143-KO
replicate 1
replicate 2
ZNF143-related (N=27,801)
ZNF143-unrelated (N=4,196)
B
CTCF, wild-type
Viny et al.
Ochi et al.
C
CTCF motif scores
normalised motif z-score
D
Fraction of peaks overlapping CISTROME CTCF peaks
ZNF143 related
ZNF143 unrelated
E
Read count, log2(observed/expected)
WT, replicate 1
WT, replicate 2
ZNF143-KO, replicate 1
ZNF143-KO, replicate 2
GC content, %
F
Zhou et al.
WT, replicate 1
WT, replicate 2
ZNF143-KO, replicate 1
ZNF143-KO, replicate 2
ZNF143-related
ZNF143-unrelated
Ochi et al.
Viny et al.
GC content
chr14: 20,700,000-20,737,000
G
ZNF143, Zhang et al.
wild-type
CTCF depletion
ZNF143-only (N=15,377)
Shared (N=19,745)
H
Zhang et al.
WT
CTCF depletion
ZNF143-only
Shared
chr12: 3,900,000-4,008,000
I
10517
ZNF143-CTCF motif pairs located 37bp apart
362406
SINE/B2 repeats
27
J
CTCF, Zhou et al. wild-type
ZNF143, Zhou et al. wild-type
replicate 1
replicate 2
ZNF143-CTCF motif pairs located 37 bp apart

Figure S4. Re-analysis of previously published data suggests that CTCF-ZNF143 binding interdependency is likely an artefact, related to Figure 3. (A) Tornado plots of CTCF ChIP-seq signal from two biological replicates in wild-type (WT) and ZNF143-knockout (KO) haematopoietic stem and progenitor cells (HSPC) centred at ZNF143-related (top) and ZNF143-unrelated (bottom) CTCF peaks[S7]. **(B)** Same as in (A) but for the CTCF ChIP-seq signal in HSPC from two orthogonal studies[S8,9]. **(C)** Rolling mean of the normalised CTCF motifs scores, annotated for the ZNF143-related (top) and ZNF143-unrelated (bottom) CTCF peaks. **(D)** Violin plots showing the fraction of ZNF143-related (left) and ZNF143-unrelated (right) CTCF peaks overlapping CTCF peaks from the CISTROME database[S10]. **(E)** GC bias scores calculated for CTCF ChIP-seq data generated from WT and ZNF143-KO HSPC samples[S7]. Note the divergence of the first WT CTCF replicate from the rest of the samples. **(F)** Genomic tracks showing CTCF ChIP-seq signal from two biological replicates in WT and ZNF143-KO HSPC[S7], CTCF ChIP-seq signal from two other HSPC samples[S8,9], and GC content. Horizontal bars indicate ZNF143-related and ZNF143-unrelated CTCF peaks[S7]. Note the overlap of ZNF143-related peaks with GC-rich regions. **(G)** Tornado plots of ZNF143 ChIP-nexus signal from control and CTCF-depleted HEC1B cells centred at ZNF143-only (top) and shared ZNF143 and CTCF (bottom) peaks[S11]. **(H)** Genomic tracks showing ZNF143 ChIP-nexus signal from control and CTCF-depleted HEC1B cells[S11]. Horizontal bars indicate ZNF143-only and shared ZNF143 and CTCF peaks. Note the specific loss of signal at shared peaks upon CTCF depletion. **(I)** Venn diagram showing the overlap between ZNF143-CTCF motif pairs located 37 bp apart from each other[S7] and SINE/B2 repeat elements in the mouse genome from RepeatMasker. **(J)** Tornado plots of CTCF and ZNF143 ChIP-seq signal centred at ZNF143-CTCF motif pairs located 37 bp apart from each other[S7].

Figure S5

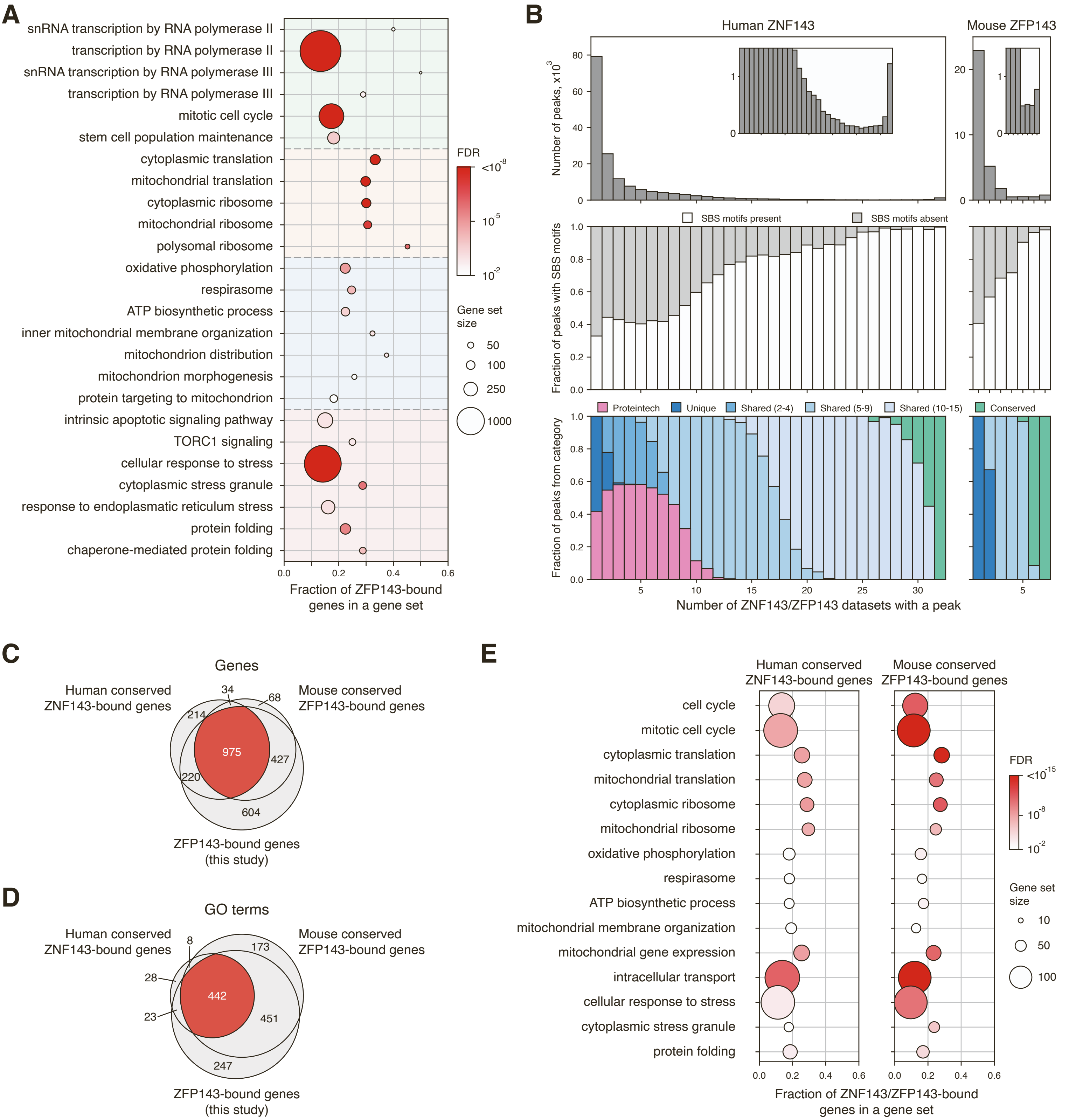
A
snRNA transcription by RNA polymerase II
transcription by RNA polymerase II
snRNA transcription by RNA polymerase III
transcription by RNA polymerase III
mitotic cell cycle
stem cell population maintenance
cytoplasmic translation
mitochondrial translation
cytoplasmic ribosome
mitochondrial ribosome
polysomal ribosome
oxidative phosphorylation
respirasome
ATP biosynthetic process
inner mitochondrial membrane organization
mitochondrion distribution
mitochondrion morphogenesis
protein targeting to mitochondrion
intrinsic apoptotic signaling pathway
TORC1 signaling
cellular response to stress
cytoplasmic stress granule
response to endoplasmatic reticulum stress
protein folding
chaperone-mediated protein folding
0.0
0.2
0.4
0.6
Fraction of ZFP143-bound genes in a gene set
FDR
$<10^{-8}$
10^{-5}
10^{-2}
Gene set size
50
100
250
1000
B
Human ZNF143
Mouse ZFP143
Number of peaks, $x10^{3}$
80
60
40
20
0
1
0
SBS motifs present
SBS motifs absent
Fraction of peaks with SBS motifs
1.0
0.8
0.6
0.4
0.2
0.0
Proteintech
Unique
Shared (2-4)
Shared (5-9)
Shared (10-15)
Conserved
Fraction of peaks from category
5
10
15
20
25
30
Number of ZNF143/ZFP143 datasets with a peak
C
Genes
Human conserved ZNF143-bound genes
Mouse conserved ZFP143-bound genes
34
68
214
975
427
220
604
ZFP143-bound genes (this study)
D
GO terms
Human conserved ZNF143-bound genes
Mouse conserved ZFP143-bound genes
8
173
28
442
23
451
247
ZFP143-bound genes (this study)
E
Human conserved ZNF143-bound genes
Mouse conserved ZFP143-bound genes
cell cycle
mitotic cell cycle
cytoplasmic translation
mitochondrial translation
cytoplasmic ribosome
mitochondrial ribosome
oxidative phosphorylation
respirasome
ATP biosynthetic process
mitochondrial membrane organization
mitochondrial gene expression
intracellular transport
cellular response to stress
cytoplasmic stress granule
protein folding
FDR
$<10^{-15}$
10^{-8}
10^{-2}
Gene set size
10
50
100
Fraction of ZNF143/ZFP143-bound genes in a gene set

Figure S5. Conservation of ZNF143 targets across cell types and organisms, related to Figure 4. (A) Gene ontology (GO) terms, overrepresented in ZFP143-bound genes, identified based on ZFP143-HA ChIP-seq data in ZFP143-FKBP cells. Coloured areas represent gene sets involved in various cellular functions. **(B)** Bar plots showing the number of ZNF143/ZFP143 peaks overlapping between datasets (top panels), the fraction of ZNF143/ZFP143 peaks with SBS motifs present (middle panels), and the number of cell types sharing ZNF143/ZFP143 peaks (bottom panels) in the re-analysed publicly available human and mouse ChIP-seq datasets. **(C)** Venn diagram showing the overlap between conserved ZNF143-bound genes in human, conserved ZFP143-bound genes in mouse, and ZFP143-bound genes identified in ZFP143-FKBP cells. **(D)** Venn diagram showing the overlap between GO terms significantly overrepresented in conserved ZNF143-bound genes in human, conserved ZFP143-bound genes in mouse, and ZFP143-bound genes identified in ZFP143-FKBP cells. **(E)** GO terms, overrepresented in conserved ZNF143/ZFP143-bound human (left panel) and mouse (right panel) genes.

Figure S6

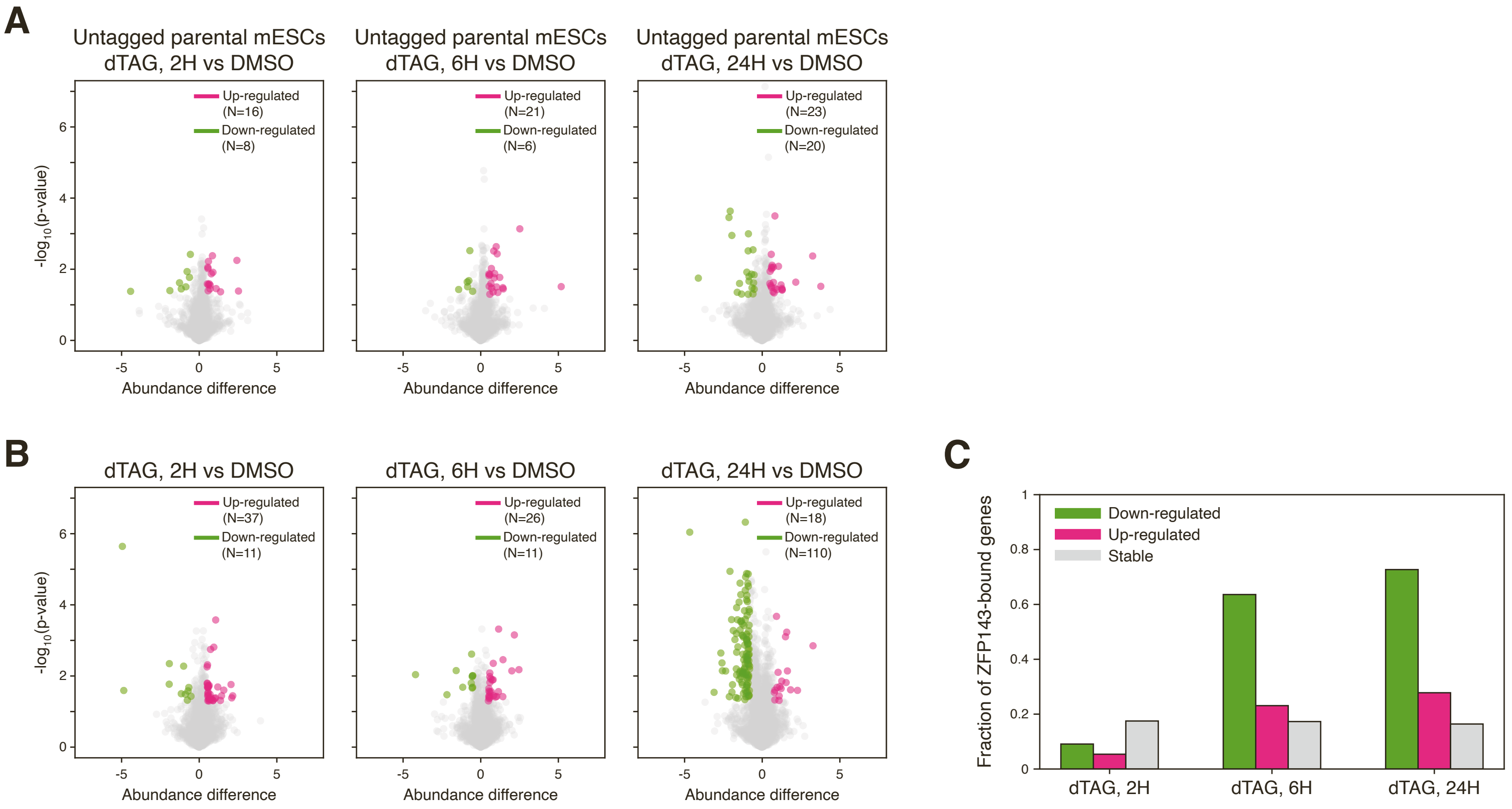

A
Untagged parental mESCs dTAG, 2H vs DMSO
Untagged parental mESCs dTAG, 6H vs DMSO
Untagged parental mESCs dTAG, 24H vs DMSO
Up-regulated (N=16)
Down-regulated (N=8)
Up-regulated (N=21)
Down-regulated (N=6)
Up-regulated (N=23)
Down-regulated (N=20)
-log10(p-value)
Abundance difference
B
dTAG, 2H vs DMSO
dTAG, 6H vs DMSO
dTAG, 24H vs DMSO
Up-regulated (N=37)
Down-regulated (N=11)
Up-regulated (N=26)
Down-regulated (N=11)
Up-regulated (N=18)
Down-regulated (N=110)
-log10(p-value)
Abundance difference
C
Fraction of ZFP143-bound genes
Down-regulated
Up-regulated
Stable
dTAG, 2H
dTAG, 6H
dTAG, 24H

Figure S6. Proteome changes after ZFP143 depletion are delayed but consistent with changes in the nascent transcriptome, related to Figure 4. (A) Volcano plot showing effect sizes and significance of the down-regulated (green) and up-regulated (pink) proteins measured by quantitative mass spectrometry in untagged E14 mESCs after dTAG-V1 treatment, compared to DMSO treatment. The number of differentially expressed proteins is indicated in the top right corners. **(B)** Same as (A) but for the ZFP143-FKBP cells. **(C)** Fraction of ZFP143-bound genes among down-regulated (green), up-regulated (pink), and stable (grey) proteins in the mass spectrometry after ZFP143 depletion.

Figure S7

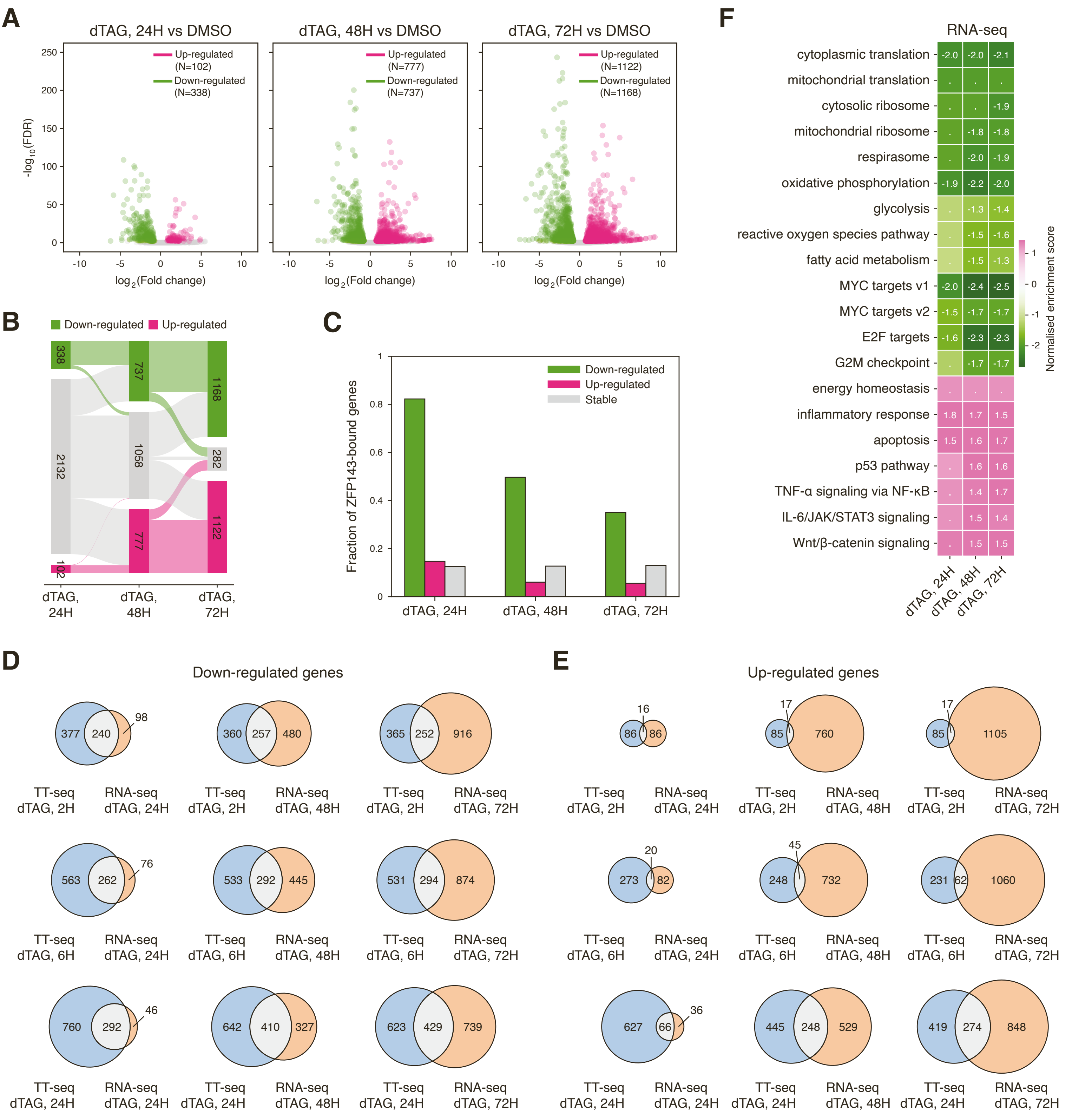
A
dTAG, 24H vs DMSO
dTAG, 48H vs DMSO
dTAG, 72H vs DMSO
Up-regulated (N=102)
Down-regulated (N=338)
Up-regulated (N=777)
Down-regulated (N=737)
Up-regulated (N=1122)
Down-regulated (N=1168)
-log10(FDR)
log2(Fold change)
B
Down-regulated
Up-regulated
338
2132
102
737
1058
777
1168
282
1122
dTAG, 24H
dTAG, 48H
dTAG, 72H
C
Fraction of ZFP143-bound genes
Down-regulated
Up-regulated
Stable
dTAG, 24H
dTAG, 48H
dTAG, 72H
F
RNA-seq
cytoplasmic translation
mitochondrial translation
cytosolic ribosome
mitochondrial ribosome
respirasome
oxidative phosphorylation
glycolysis
reactive oxygen species pathway
fatty acid metabolism
MYC targets v1
MYC targets v2
E2F targets
G2M checkpoint
energy homeostasis
inflammatory response
apoptosis
p53 pathway
TNF-α signaling via NF-κB
IL-6/JAK/STAT3 signaling
Wnt/β-catenin signaling
Normalised enrichment score
dTAG, 24H
dTAG, 48H
dTAG, 72H
D
Down-regulated genes
TT-seq dTAG, 2H
TT-seq dTAG, 6H
TT-seq dTAG, 24H
RNA-seq dTAG, 24H
RNA-seq dTAG, 48H
RNA-seq dTAG, 72H
E
Up-regulated genes

Figure S7. Changes in the mRNA levels following ZFP143 depletion are consistent with nascent RNA production, related to Figure 4. (A) Volcano plots showing effect sizes and significance of the down-regulated (green) and up-regulated (pink) genes measured by RNA-seq after dTAG-V1 treatment, compared to DMSO treatment. The number of differentially expressed genes is indicated in the top right corners. **(B)** Sankey diagram showing the differentially expressed genes following ZFP143 depletion: up-regulated (pink) and down-regulated (green) genes at FDR < 0.05, absolute log2-fold-change > 0.5. **(C)** Fraction of ZFP143-bound genes among down-regulated (green), up-regulated (pink), and stable (grey) genes in the RNA-seq after ZFP143 depletion. **(D)** Venn diagram showing the overlap between down-regulated genes detected in TT-seq and RNA-seq following the time course of ZFP143 depletion. **(E)** Same as in (D) but for up-regulated genes. **(F)** Gene set enrichment analysis of RNA-seq data after ZFP143 depletion (up-regulated gene sets in pink, down-regulated gene sets in green). The normalised enrichment score for significant gene sets (FDR < 0.1) is shown.

Figure S8

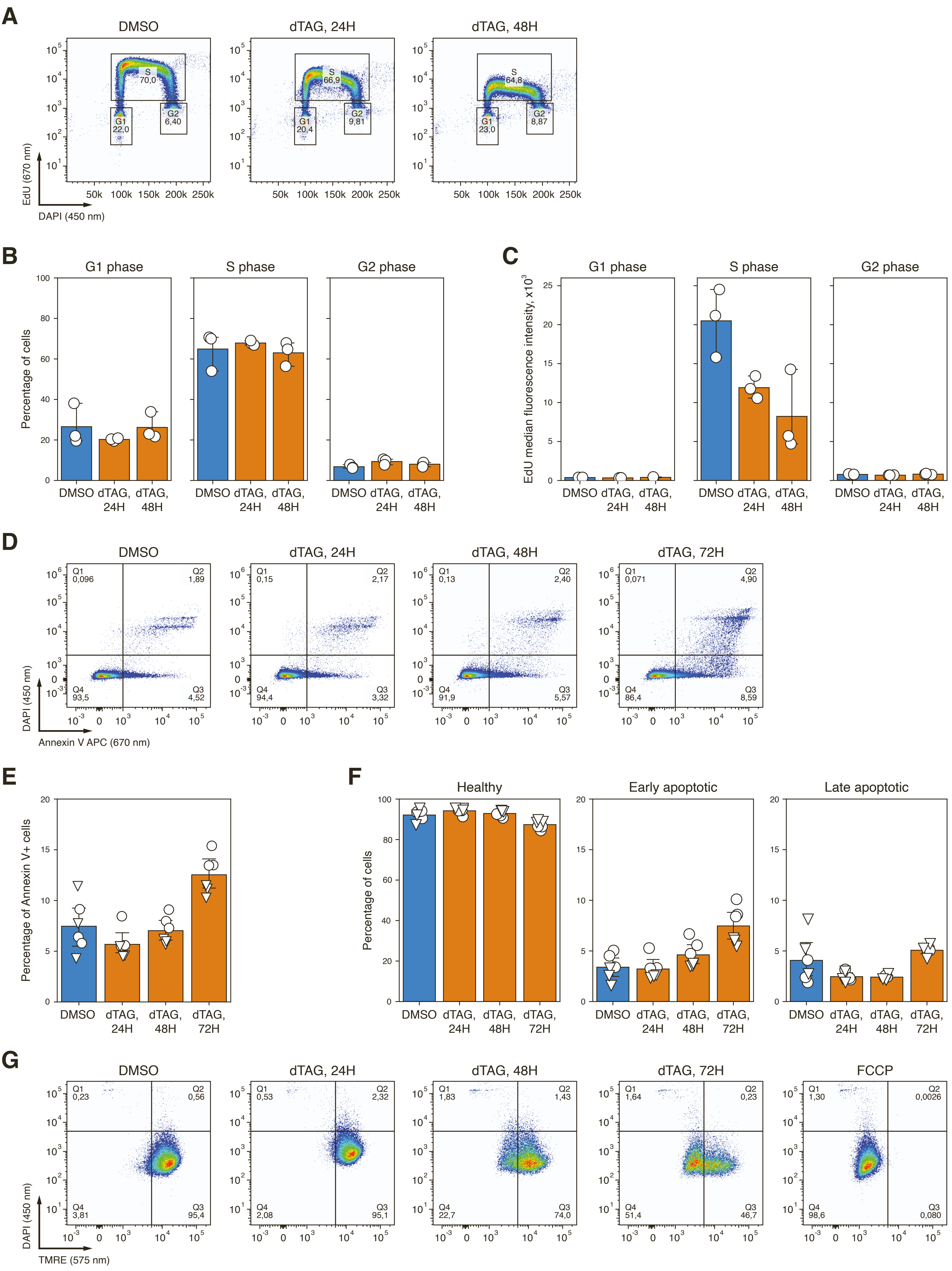

A
DMSO
dTAG, 24H
dTAG, 48H
S
70,0
66,9
64,8
G1
22,0
20,4
23,0
G2
6,40
9,81
8,87
EdU (670 nm)
DAPI (450 nm)
50k 100k 150k 200k 250k
B
G1 phase
S phase
G2 phase
Percentage of cells
DMSO dTAG, 24H dTAG, 48H
C
G1 phase
S phase
G2 phase
EdU median fluorescence intensity, x10^3
DMSO dTAG, 24H dTAG, 48H
D
DMSO
dTAG, 24H
dTAG, 48H
dTAG, 72H
Q1 0,096 Q2 1,89 Q4 93,5 Q3 4,52
Q1 0,15 Q2 2,17 Q4 94,4 Q3 3,32
Q1 0,13 Q2 2,40 Q4 91,9 Q3 5,57
Q1 0,071 Q2 4,90 Q4 86,4 Q3 8,59
DAPI (450 nm)
Annexin V APC (670 nm)
E
Percentage of Annexin V+ cells
DMSO dTAG, 24H dTAG, 48H dTAG, 72H
F
Healthy
Early apoptotic
Late apoptotic
Percentage of cells
DMSO dTAG, 24H dTAG, 48H dTAG, 72H
G
DMSO
dTAG, 24H
dTAG, 48H
dTAG, 72H
FCCP
Q1 0,23 Q2 0,56 Q4 3,81 Q3 95,4
Q1 0,53 Q2 2,32 Q4 2,08 Q3 95,1
Q1 1,83 Q2 1,43 Q4 22,7 Q3 74,0
Q1 1,64 Q2 0,23 Q4 51,4 Q3 46,7
Q1 1,30 Q2 0,0026 Q4 98,6 Q3 0,080
DAPI (450 nm)
TMRE (575 nm)

Figure S8. Flow cytometry analysis of cell cycle, apoptosis, and mitochondrial membrane potential following ZFP143 loss, related to Figure 5. (A) Representative flow cytometry data plots for EdU incorporation assay to measure cell cycle phases in DMSO and dTAG-V1 treated cells. **(B)** Quantification of cell cycle phases in DMSO (blue) and dTAG-V1 (orange) treated cells. Dots represent values for replicates. Error bars indicate 95% confidence interval. **(C)** Quantification of EdU median fluorescence intensity for different cell cycle phases in DMSO (blue) and dTAG-V1 (orange) treated cells. Dots represent values for replicates. Error bars indicate 95% confidence interval. **(D)** Representative flow cytometry data plots for Annexin V staining to measure apoptosis rates in DMSO and dTAG-V1 treated cells. **(E)** Quantification of total Annexin V-positive cells in DMSO (blue) and dTAG-V1 (orange) treated cells. Dots represent values for replicates. Error bars indicate 95% confidence interval. **(F)** Quantification of healthy, early apoptotic and late apoptotic cells in DMSO (blue) and dTAG-V1 (orange) treated cells. Dots represent values for replicates. Error bars indicate 95% confidence interval. **(G)** Representative flow cytometry data plots for TMRE staining to measure mitochondrial membrane potential in DMSO, dTAG-V1, and FCCP treated cells.

Figure S9

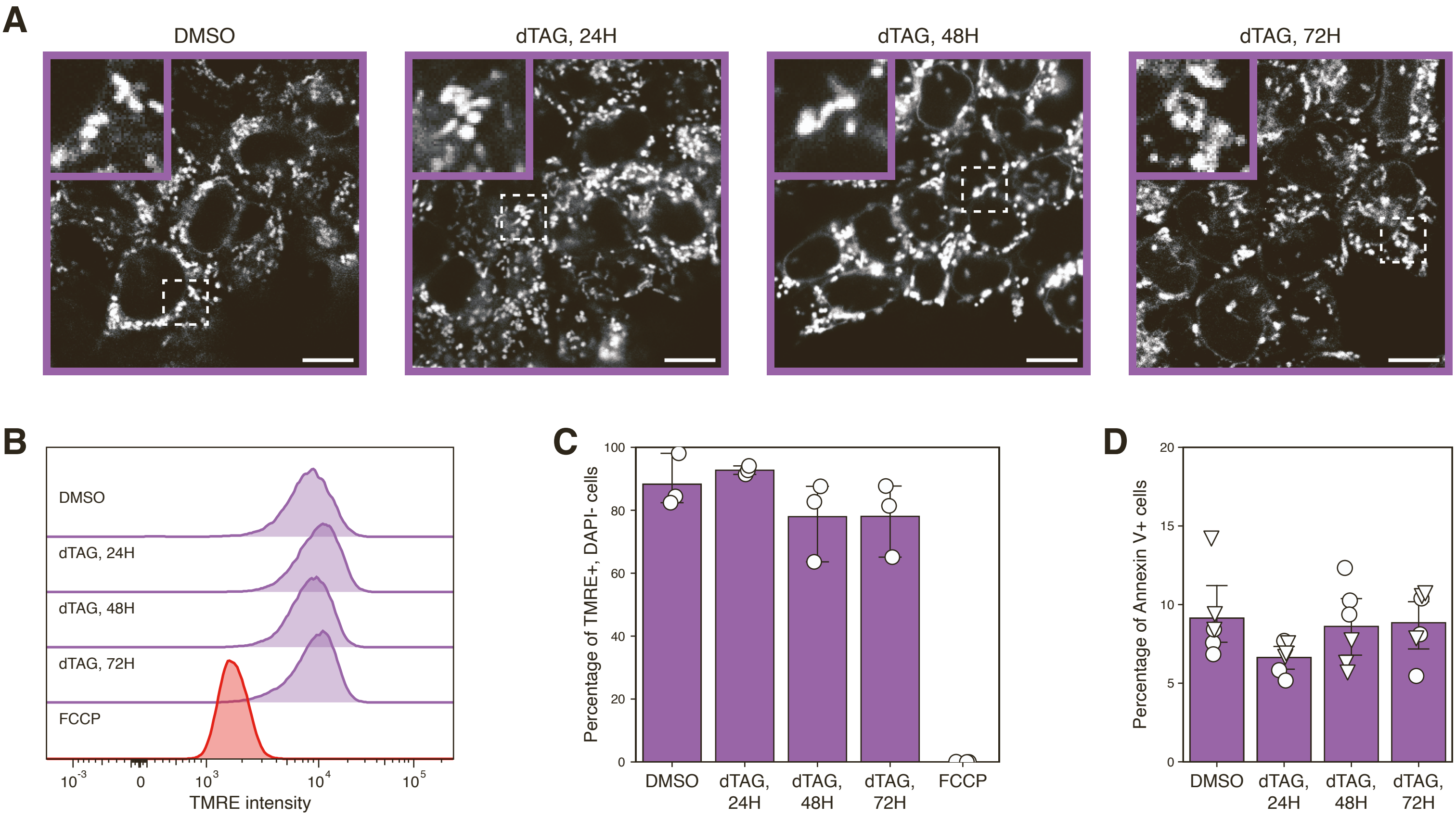

A
DMSO
dTAG, 24H
dTAG, 48H
dTAG, 72H
B
DMSO
dTAG, 24H
dTAG, 48H
dTAG, 72H
FCCP
10^{-3}
0
10^{3}
10^{4}
10^{5}
TMRE intensity
C
Percentage of TMRE+, DAPI- cells
100
80
60
40
20
0
DMSO
dTAG, 24H
dTAG, 48H
dTAG, 72H
FCCP
D
Percentage of Annexin V+ cells
20
15
10
5
0
DMSO
dTAG, 24H
dTAG, 48H
dTAG, 72H

Figure S9. Treatment of untagged E14 mESCs with dTAG-V1 does not affect mitochondria, related to Figure 5. (A) Representative live-cell confocal microscopy images of the MitoTracker Red FM fluorescence in DMSO and dTAG-V1 treated untagged E14 mESCs. Magnified images of mitochondrial morphology and mitochondrial network for the dash-boxed regions in the representative images are shown in the upper-left corners. Scale bar: 10 μm. **(B)** Representative flow cytometry histogram showing TMRE fluorescence distribution in DMSO (purple), dTAG-V1 (purple), and FCCP (red) treated untagged E14 mESCs. **(C)** Quantification of TMRE-positive cells in DMSO (purple), dTAG-V1 (purple), and FCCP (red) treated untagged E14 mESCs. Dots represent values for replicates. Error bars indicate 95% confidence interval. **(D)** Quantification of total Annexin V-positive cells in DMSO and dTAG-V1 treated untagged E14 mESCs. Dots represent values for replicates. Error bars indicate 95% confidence interval.

Figure S10

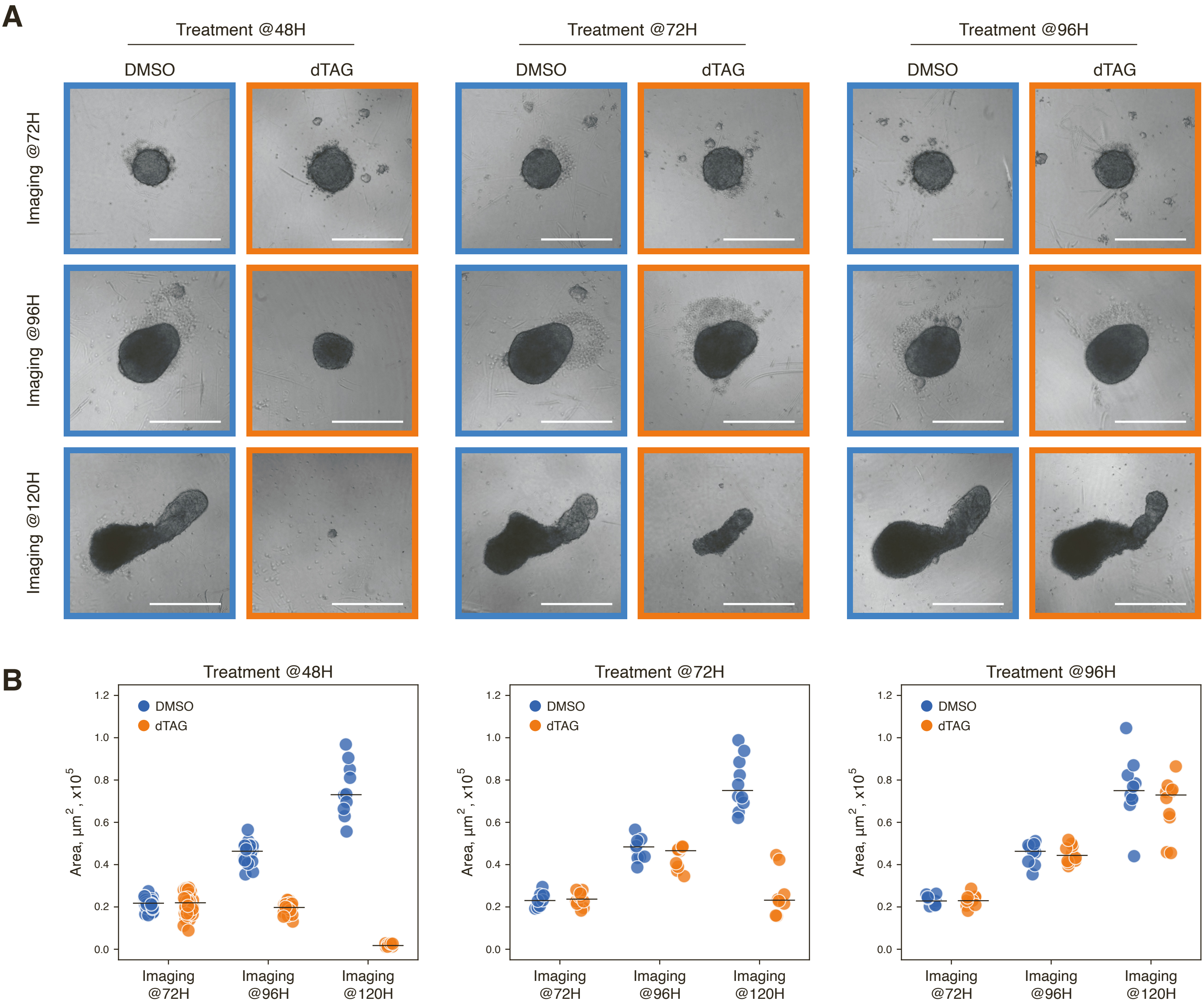
A
Treatment @48H
Treatment @72H
Treatment @96H
DMSO
dTAG
Imaging @72H
Imaging @96H
Imaging @120H
B
Treatment @48H
Treatment @72H
Treatment @96H
DMSO
dTAG
Area, µm², x10⁵
1.2
1.0
0.8
0.6
0.4
0.2
0.0
Imaging @72H
Imaging @96H
Imaging @120H

Figure S10. Time-course imaging and analysis of gastruloids morphology, related to Figure 6. (A) Bright-field microscopy images showing gastruloids treated with DMSO (blue frame) and dTAG-V1 (orange frames) at the times indicated on top and imaged at the developmental stages indicated on the left. Scale bar: 300 µm. **(B)** Quantification of gastruloid sizes for gastruloids treated with DMSO (blue) and dTAG-V1 (orange) from (A). Black lines indicate the mean values. The number of quantified gastruloids is indicated below.

Figure S11

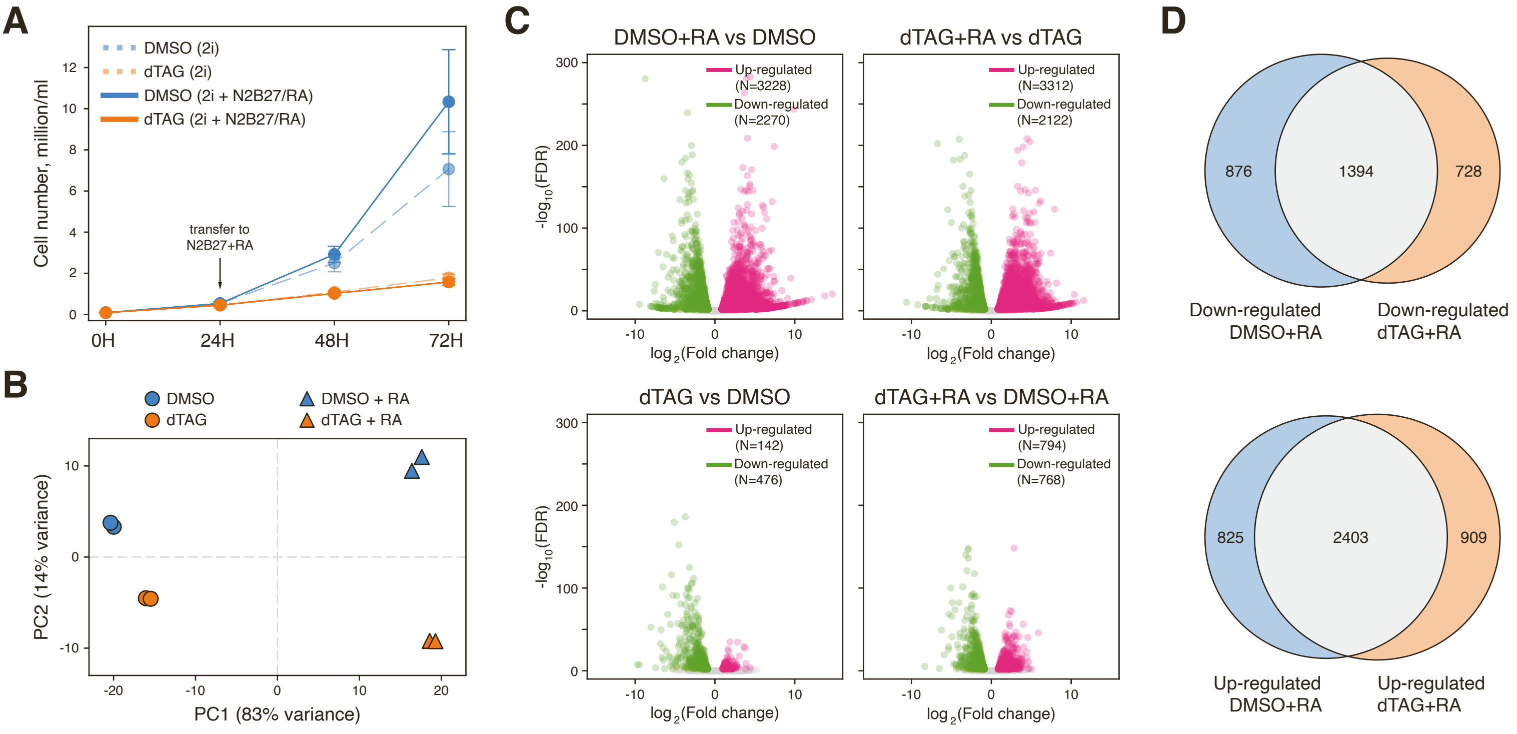
A
DMSO (2i)
dTAG (2i)
DMSO (2i + N2B27/RA)
dTAG (2i + N2B27/RA)
Cell number, million/ml
transfer to N2B27+RA
0H
24H
48H
72H
B
DMSO
dTAG
DMSO + RA
dTAG + RA
PC2 (14% variance)
PC1 (83% variance)
C
DMSO+RA vs DMSO
Up-regulated (N=3228)
Down-regulated (N=2270)
dTAG+RA vs dTAG
Up-regulated (N=3312)
Down-regulated (N=2122)
dTAG vs DMSO
Up-regulated (N=142)
Down-regulated (N=476)
dTAG+RA vs DMSO+RA
Up-regulated (N=794)
Down-regulated (N=768)
-log10(FDR)
log2(Fold change)
D
876
1394
728
Down-regulated DMSO+RA
Down-regulated dTAG+RA
825
2403
909
Up-regulated DMSO+RA
Up-regulated dTAG+RA

Figure S11. Transcriptomic changes upon differentiation with retinoic acid, related to Figure 6. (A) Growth curve showing the total cell number of DMSO (blue) and dTAG-V1 (orange) treated cells grown in 2i media (dashed line) and after transfer to N2B27 differentiation media with retinoic acid 24 hours post-depletion start (solid line). Dots indicate mean values. Error bars indicate standard deviation. **(B)** Principal component analysis of the RNA-seq data of DMSO (blue) and dTAG-V1 (orange) treated cells grown in 2i media (circles) and after transfer to N2B27 differentiation media with retinoic acid 24 hours post-depletion start (triangles). **(C)** Volcano plot showing effect sizes and significance of the down-regulated (green) and up-regulated (pink) genes measured by RNA-seq before and after retinoic acid treatment in DMSO and dTAG-V1 treated cells. Top row shows volcano plots for the effect of retinoic acid in DMSO (left panel) and dTAG-V1 (right panel) treated cells. Bottom row shows volcano plots for the effect of dTAG-V1 treatment before (left panel) and after (right panel) transfer to differentiation media. The number of differentially expressed genes is indicated in the top right corners. **(D)** Venn diagram showing the overlap between down-regulated (top panel) and up-regulated (bottom panel) genes in DMSO and dTAG-V1 treated mESCs before and after differentiation with retinoic acid.

[S1]. Pintacuda, G., Wei, G., Roustan, C., Kirmizitas, B.A., Solcan, N., Cerase, A., Castello, A., Mohammed, S., Moindrot, B., Nesterova, T.B., and Brockdorff, N. (2017). hnRNPK Recruits PCGF3/5-PRC1 to the Xist RNA B-Repeat to Establish Polycomb-Mediated Chromosomal Silencing. Mol Cell *68*, 955-969 e910. 10.1016/j.molcel.2017.11.013.
[S2]. Fornes, O., Castro-Mondragon, J.A., Khan, A., van der Lee, R., Zhang, X., Richmond, P.A., Modi, B.P., Correard, S., Gheorghe, M., Baranasic, D., et al. (2020). JASPAR 2020: update of the open-access database of transcription factor binding profiles. Nucleic Acids Res *48*, D87-D92. 10.1093/nar/gkz1001.
[S3]. Ngondo-Mbongo, R.P., Myslinski, E., Aster, J.C., and Carbon, P. (2013). Modulation of gene expression via overlapping binding sites exerted by ZNF143, Notch1 and THAP11. Nucleic Acids Res *41*, 4000-4014. 10.1093/nar/gkt088.
[S4]. Hsieh, T.S., Cattoglio, C., Slobodyanyuk, E., Hansen, A.S., Darzacq, X., and Tjian, R. (2022). Enhancer-promoter interactions and transcription are largely maintained upon acute loss of CTCF, cohesin, WAPL or YY1. Nat Genet *54*, 1919-1932. 10.1038/s41588-022-01223-8.
[S5]. Hsieh, T.S., Cattoglio, C., Slobodyanyuk, E., Hansen, A.S., Rando, O.J., Tjian, R., and Darzacq, X. (2020). Resolving the 3D Landscape of Transcription-Linked Mammalian Chromatin Folding. Mol Cell *78*, 539-553 e538. 10.1016/j.molcel.2020.03.002.
[S6]. Dong, J., Scott, T.G., Mukherjee, R., and Guertin, M.J. (2024). ZNF143 binds DNA and stimulates transcripstion initiation to activate and repress direct target genes. bioRxiv. 10.1101/2024.05.13.594008.
[S7]. Zhou, Q., Yu, M., Tirado-Magallanes, R., Li, B., Kong, L., Guo, M., Tan, Z.H., Lee, S., Chai, L., Numata, A., et al. (2021). ZNF143 mediates CTCF-bound promoter-enhancer loops required for murine hematopoietic stem and progenitor cell function. Nat Commun *12*, 43. 10.1038/s41467-020-20282-1.
[S8]. Viny, A.D., Bowman, R.L., Liu, Y., Lavallee, V.P., Eisman, S.E., Xiao, W., Durham, B.H., Navitski, A., Park, J., Braunstein, S., et al. (2019). Cohesin Members Stag1 and Stag2 Display Distinct Roles in Chromatin Accessibility and Topological Control of HSC Self-Renewal and Differentiation. Cell Stem Cell *25*, 682-696 e688. 10.1016/j.stem.2019.08.003.
[S9]. Ochi, Y., Kon, A., Sakata, T., Nakagawa, M.M., Nakazawa, N., Kakuta, M., Kataoka, K., Koseki, H., Nakayama, M., Morishita, D., et al. (2020). Combined Cohesin-RUNX1 Deficiency Synergistically Perturbs Chromatin Looping and Causes Myelodysplastic Syndromes. Cancer Discov *10*, 836-853. 10.1158/2159-8290.CD-19-0982.
[S10]. Zheng, R., Wan, C., Mei, S., Qin, Q., Wu, Q., Sun, H., Chen, C.H., Brown, M., Zhang, X., Meyer, C.A., and Liu, X.S. (2019). Cistrome Data Browser: expanded datasets and new tools for gene regulatory analysis. Nucleic Acids Res *47*, D729-D735. 10.1093/nar/gky1094.
[S11]. Zhang, M., Huang, H., Li, J., and Wu, Q. (2024). ZNF143 deletion alters enhancer/promoter looping and CTCF/cohesin geometry. Cell Rep *43*, 113663. 10.1016/j.celrep.2023.113663.
